# Integrating genetic and physical positions of the anthracnose resistance genes described in bean chromosomes Pv01 and Pv04

**DOI:** 10.1371/journal.pone.0212298

**Published:** 2019-02-14

**Authors:** Ester Murube, Ana Campa, Juan José Ferreira

**Affiliations:** Plant Genetic Group, Area of Horticultural and Forest Crops, SERIDA, Villaviciosa Asturias, Spain; Università Politecnica delle Marche, ITALY

## Abstract

A complex landscape of anthracnose resistance genes (*Co-*) located at the telomeric regions of the bean chromosomes Pv01 and Pv04 has been reported. The aim of this work was to investigate the genetic and physical positions of genes conferring resistance to races 6, 38, 39, 357, 65, and 73 as well as the relationships among the resistance genes identified herein and the previously described Co- genes in these telomeric regions. The linkage analysis using a genetic map of 497 SNPs from the recombinant inbred line population Xana/BAT93 revealed that the gene conferring resistance to race 65 in cultivar Xana (*Co-1*^*65-X*^) was located in the Co-1 cluster, at the distal end of chromosome Pv01. The fine mapping of *Co-1*^*65-X*^ indicated that it was positioned between the physical positions 49,512,545 and 49,658,821 bp. This delimited physical position agrees with the positions of the previously mapped genes *Co- 1*^*4*^, *Co-x*, *Co-14*, *Co-1*^*HY*^, and *Co-Pa*. Responses to races 6, 38, 39, and 357 in BAT93 exhibited co-segregation suggesting that the same gene, or very closely linked genes, were involved in the control. The linkage analysis showed that the resistance gene to race 38 in the genotype BAT93 (*Co-3*^*38-B*^) was located at the beginning of chromosome Pv04, in the genetic position of the Co-3 cluster, and was flanked by markers with physical positions between 1,286,490 and 2,047,754 bp. Thus, the genes Co-3, Co-9, Co-10, Co-16, and *Co-3*^*38-B*^, found in this work, form part of the same anthracnose resistance cluster at the beginning of chromosome Pv04, which is consistent with the discontinuous distribution of typical R genes annotated in the underlying genomic region. Resistance loci involved in the response to race 73 in the genotypes Xana (R) and BAT93 (R) were mapped to the same positions on clusters Co-1 and Co-3, respectively. The positioning of the resistance genes in the bean genome based on fine linkage mapping should play an important role in the characterization and differentiation of the anthracnose resistance genes. The assignment of Co- genes to clusters of race specific genes can help simplify the current scenario of anthracnose resistance.

## Introduction

Anthracnose, caused by the ascomycete fungus, *Colletotrichum lindemuthianum* (Sacc. & Magnus) Lams- Scrib., is one of many diseases that can adversely affect the common bean (*Phaseolus vulgaris* L.) [1]. Typical symptoms are deep and well delimited lesions on hypocotyls, stems, leaf veins, pods, and seeds that usually have salmon-colored spores. Disease progression is favored by humid environments with moderate temperatures and can lead to the eventual death of the plants. The fungus has a high pathogenic variability level classified into physiological races based on response profiles to a set of 12 differential common bean cultivars [2]. To date, the inoculation of ~1,590 isolates of *C*. *lindemuthianum* on this standardized cultivar set has resulted in the identification of 182 races worldwide [3].

Resistance to *C*. *lindemuthianum* in common bean is very specific, so that a resistance gene from a bean genotype confers protection against specific isolates or races. Based on results supplied by allelism tests and, in some cases, linkage analyses, many anthracnose resistance genes (named as Co-; see bean gene list 2017 in http://arsftfbean.uprm.edu/bic/wp-content/uploads/2018/04/BeanGenesList2017.pdf, visited on September 2018) have been identified and located on chromosomes Pv01, Pv02, Pv03, Pv04, Pv07, Pv08, and Pv11 [4, 5]. Most genes show complete dominance, although a few genes with a complementary mode of action were also identified [6, 7]. Nevertheless, mapping of genes conferring resistance to specific isolates or races revealed that the *Co*- genes were organized in clusters of very close race-specific resistance genes that undergo recombination among themselves (e.g., cluster Co-3 on Pv04, cluster Co-5 on Pv07, and cluster Co-2 on Pv11) [6, 8, 9]. This organization in clusters is consistent with the occurrence in the bean genome of clusters of *R* genes with leucine-rich repeat (LRR) or protein kinase (PK) domains which are typically involved in the resistance response [10]. These R gene clusters underly genetic positions of the anthracnose resistance genes [11].

Many studies have located anthracnose resistance genes in the telomeres of bean chromosomes Pv01 and Pv04. In fact, these regions were detected in a genome-wide association study (GWAS) conducted for the reactions of 235 Andean lines to eight *C*. *lindemuthianum* races [12]. At the distal end of Pv01, the *Co-1* gene (originally known as gene A) was proposed to be involved in the resistance to race 73 in the cultivar MDRK on the LG Pv01 [7]. A gene conferring resistance to race 1545 in MDRK was linked to the marker OF10_530_, which mapped in LG Pv01 [13, 14]. Based on resistance spectra observed in different bean genotypes and the positions in the bean genetic map, five alleles have been reported for the locus *Co-1*: *Co-1*^*2*^ in Kaboon; *Co-1*^*3*^ in Perry Marrow [7]; *Co-1*^*4*^, flanked by the markers CV542014 and TGA1.1 in AND277 [15]; *Co-1*^*5*^, linked in repulsion phase to marker OA18^1500^ in Widusa [16]; and *Co-1*^*HY*^, flanked by the markers PSSR0869 and PSSR0771 in the genotype Honyundou [17]. Furthermore, three resistance genes, without evidence of being allelic to *Co-1*, were located at the end of chromosome Pv01: *Co-x*, identified in vicinity of the marker CV542014 in the cultivar JaloEEP558 [18, 19]; *Co-Pa*, identified in cultivar Paloma and flanked by SNP markers SS82 and SS83 [20]; and *Co-14* identified in cultivar Pitanga and linked to the marker CV542014 at 4.3 cM [21, 22]. Three race specific genes to races 65, 73, and 81 (named as *Co-1*^*73-X*^, *Co-1*^*65-X*^, *R*^*81*^) in the genotypes Xana and Kaboon were also located in this genetic position [6, 23].

At the proximal end of Pv04, the *Co-3* gene (or *Mexique 1*) was described in the cultivar Mexico 222 using the isolates 14 and 51 [24], and later, two race-specific resistance genes to races 19 and 31 (R^19^ and R^31^) from Mexico 222 were mapped on LG Pv04 between the markers Pv-ctt001 and SW12^700^ [9]. Four alleles were proposed for the *Co-3* locus based on the resistance spectrum; *Co-3*^*2*^ in the cultivar Mex227, which is currently not available [25]; *Co-3*^*3*^ (first named as Co-9) from BAT93 linked to markers 254-G15F and GA-G16 [26]; *Co-3*^*4*^ (first named a *Co-10*) in cultivar Ouro Negro linked to marker g2303 and flanked by KASP152 and KASP153 [27, 28, 29]; and *Co-3*^*5*^, previously identified as *Co-7* in G2333 [30, 31]. Other *Co-*genes were mapped in chromosome Pv04: the closely linked genes *Co-z* and *Co-y* from the genotype JaloEEP558 linked to D1174 [18, 32]; *Co-15* from Corinthiano that is linked to marker g2685 [33]; and the *Co-16* gene reported in the cultivar Crioulo 159 as linked to marker g2467 [34]. Moreover, analyzing the segregating population obtained from the cross Mexico 222 × Widusa, four linked race-specific resistance genes from Widusa (R^65^, R^73^, R^102^ and R^449^) were mapped on Pv04 between the markers Pv-ctt001 and SW12^700^ [9]. Finally, evidence of resistance genes to races 3, 7, 19, 449, and 453, showing a complementary mode of action, were detected in this same genetic position in the cultivar Xana [6].

Genetic studies based on allelism tests or linkage analyses in different bean genotypes have led to a complex scenario in which different anthracnose resistance genes have been reported in similar genetic positions even though the organization of anthracnose race-specific gene clusters has been well established [6, 8, 9]. The annotated bean genome is now available (www.phytozome.net), and an accurate physical position in the genome of candidate resistance genes can help clarify the complex structure of the resistance loci located on LG Pv01 and Pv04. The main aim of this work was to investigate the genetic and physical positions of genes conferring resistance to races 6, 38, 39, 357, 65, and 73 in a recombinant inbred line population derived from the cross Xana × BAT93, two bean genotypes with anthracnose resistance genes in the telomeric regions of the bean chromosomes Pv01 and Pv04 [6, 11]. The resistance to races 6, 38, 39, and 357 were not previously investigated in the genotype BAT 93. Using the bean genome sequence, the relationships among the resistance genes identified herein and the previously described *Co-* genes were also investigated.

## Material and methods

### Plant material

A total of 145 F_2:6_ recombinant inbred lines (RILs) developed from the cross Xana x BAT93 was used (XB population). The population was obtained by single seed descent method from individual F_2_ plants. BAT93 is a breeding line developed at the Centro Internacional de Agricultura Tropical (CIAT), Colombia from a double cross involving four Middle American genotypes (Veranic 2, PI 207262, Jamapa, and Great Northern Tara). BAT93 has small tan or beige seeds (24 g/100 seed), indeterminate postrate growth habit and posseses resistance to several diseases [35]. BAT93 has resistance genes to anthracnose, *Co-u* and *Co-9*, located in the telomere of Pv02 and Pv04, respectively [32]. Xana is a breeding line developed from a cross between the two Andean landraces, Andecha and V203, at Servicio Regional de Investigación y Desarrollo Agroalimentario (SERIDA), Spain. Xana has a very large (100 g/100 seeds) white seeds with determinate growth habit and possesses several anthracnose resistance genes some of them located on the distal end of Pv01 [6].

The 12 common bean differential cultivars proposed for the classification in races of *C*. *lindemuthianum* pathogenic variability [2] were used as controls and to confirm the identity of each anthracnose race.

### Inoculation procedure and disease scoring

Six *C*. *lindemuthianum* isolates classified as different races were used in this study: races 39, 65 (from Brazil), 73, and 357 from the collection of the Crop and Soil Sciences Department (Michigan State University, USA), and races 6 and 38 from the SERIDA collection. All isolates were obtained from monosporic cultures maintained in fungus-colonized filter paper at –18°C for long-term storage. To obtain abundant spores, the isolates were grown at 21°C in darkness for 10 days in potato dextrose agar (DIFCO, Becton Dickinson and Company, Sparks, MD, USA). Spore suspensions were prepared by flooding the plates with 5 ml of 0.01% Tween 20 (Sigma-Aldrich, St. Louis, MO, USA) in sterile distilled water and scraping the surface of the culture with a spatula. Inoculations were carried out by spraying 8–10 days-old seedlings with a spore suspension containing 1.2 × 10^6^ spores/ml. The seedlings were maintained in a climate chamber at 20–22°C, 95–100% humidity and 12-h photoperiod. Responses of the plants were evaluated after 7–9 days. Seedlings with no visible symptoms or showing very small lesions on leaves and stems were considered resistant (R), while seedlings with large sporulation lesions or death were considered susceptible (S). The response to a specific race was evaluated by inoculating all recombinant lines in the same test, including at least 8 seedlings per line. Parental lines Xana and BAT93 and 12 common bean anthracnose differential cultivars were also included in each test as controls. RILs showing resistant and susceptible seedlings against the same race were considered heterozygous for the resistance gene. In order to show the specific interaction *C*. *lindemuthianum* genotype- *P*. *vulgaris* genotype, the resistance genes were tentatively named in this study considering the relative position on the anthracnose resistance clusters (Co- cluster), the name of the *C*. *lindemuthianum* race followed by the bean genotype in which the resistance gene was identified (in superscript) [4].

### DNA isolation and Genotyping by sequencing analysis (GBS)

Genomic DNA was isolated from young leaves of individual plants (F_6_ or F_7_) using the CTAB method described by Doyle and Doyle [36]. Concentrations of DNA were quantified photometrically (absorbance measurements between 260 and 280 nm) using a Biomate 3 ultraviolet–visible spectrophotometer (Thermo Scientific, Waltham, Massachusetts, USA). The quality levels of DNA samples were verified in 1% agarose gels, stained RedSafe (INtRON, Biotechnology, Gyunggi-Do, Korea) and visualized under ultraviolet light. The DNA samples were preserved at—80°C.

GBS, as described by Elshire et al. [37], was performed at the Institute of Genomic Diversity, (Cornell University, Ithaca, NY, USA). Briefly, DNA was digested individually with the *Ape*KI restriction enzyme, which recognizes a five base pair sequence (GCWGC, where W can be either A or T). One 95-plex GBS sequencing library was prepared by ligating the digested DNA to unique nucleotide adapters (barcodes) followed by PCR with flow-cell attachment site tagged primers. Sequencing was performed using Illumina HiSeq2000. The sequencing reads from different genotypes were de-convoluted using the barcodes and aligned to the *Phaseolus vulgaris* L. reference genome (available at www.phytozome.net) using the Burrow Wheelers Alignment tool [38]. The bean genome version was recently updated combining a new PacBio-based assembly of the genome with a re-annotation using the GMI (Gene Model Improvement) pipeline. SNPs were extracted using the GBS pipeline implemented in TASSEL 5.0 software [39].

### Molecular marker analyses

To develop a fine map around candidate regions for the resistance genes and investigate the relationship with previously anthracnose resistance genes mapped in similar genetic positions, an expanded set of molecular markers was also analyzed: i) markers previously linked to anthracnose resistance genes located on Co-1 and Co-3 clusters [for more details see S1 Table]; ii) InDel markers (Insertion-deletion polymorphisms), based on their physical positions on the bean genome [40]; iii) microsatellites specifically designed from the *P*.*vulgaris* genomic sequence using the software BatchPrimer3 and Oligo Anlyzer [41].

PCR amplification was performed in a Verity Thermal Cycler (Applied Biosystems, Life Technologies, CA, USA) in a final volume of 20 μL solution containing 25 ng of genomic DNA, 100 mM Tris–HCl, 100 mM KCl (pH 8.3), 4 mM MgCl2, 0.2 mM each dNTP (Bioline, London, UK), 0.2 μM each primer, and 1.25 U of Biotaq DNA polymerase (Bioline). Amplification products were resolved on polyacrylamide (8%) or agarose gels (1.5%) in 1x TBE buffer (89 mM TRIS, 89 mM boric acid, 2 mM EDTA), stained with RedSafe and visualized under UV light.

### Genetic analysis

To locate the anthracnose resistance genes, a genetic linkage map was developed for the XB RIL population. SNPs with heterozygous genotype, no more than 10% missing data, physical distance < 0.1Mb and, significant deviation from the expected Mendelian segregation ratio (1:1) as determined by chi-square analysis (α < 0.05), were removed. Linkage analysis was performed with the OneMap package in R [42, 43] using a log of likelihood ratio threshold (LOD) of 4.0 and a maximum recombination fraction (RF) of 0.30. Loci order was estimated based on rapid chain delineation algorithm [44] and ripple analyses. Distances between ordered loci (in cM) were calculated using the Kosambi mapping function. For SNPs forming blocks without observed recombination among them (redundant SNPs), only one SNP was used for map generation. The linkage map was drawn with the help of the software MapChart 2.32 [45].

When observed segregation suggested the presence of more than one resistance gene, a genetic dissection was carried out as described by Campa et al. [6]. First, candidate chromosome regions to be involved in the resistance response were identified by contingency chi-square tests of the joint segregation for each scored resistance with genotype for each marker included on the XB linkage map. Significant deviation from random segregation (after apply Bonferroni correction) suggests that the chromosome region tagged with the marker could be involved in the resistance response. To confirm this hypothesis, two subpopulations were established for each candidate chromosome region from the original RIL population considering the parental genotype (Xana or BAT93) for the molecular markers tagging each region. If the resistance gene is located in the region tagged changes in the segregation ratio compared to that of the total XB population are expected in the subpopulations. In the subpopulations with monogenic segregation for the resistance, a linkage analysis was performed in order to locate putative resistance gene.

### Bean genome exploration

The physical positions of the reported markers were estimated by aligning of the sequences of the specific primers or the amplicon (obtained from http://phaseolusgenes.bioinformatics.ucdavis.edu/, accessed on September 2018) with the two available P. vulgaris genomes (G19833 and BAT93) using BLAST (Basic Local Alignment Search Tool) algorithm. Finally, we took advantage of the two bean genomes of P. vulgaris [46, 47] to investigate the annotations of the predicted genes within the delimited genomic regions (G19833 in https://phytozome.jgi.doe.gov/pz/portal.html/; BAT93 in https://genomevolution.org/coge/; verified september 2018).

## Results

### GBS analysis and linkage map construction

Sequencing of the GBS libraries yielded ~381 million reads, resulting in 3,081,481 tags after merging, with 71.7% aligned to unique positions. After considering data completeness (90%) and homozygous genotypes, there were 7,145 SNPs or GBS polymorphic sites identified between the parental lines Xana and BAT93. A total of 1,555 SNPs were discarded due to distorted segregation (χ^2^; *ƿ* < 0.05). Finally, 5,590 segregating SNPs were used to construct the genetic linkage map. The final version of the XB linkage map contained 497 informative SNPs (showing recombination) distributed along the 11 linkage groups corresponding with the 11 bean chromosomes (S2 Table and S1 Fig). The number of SNPs that conformed to each LG ranged from 76 SNP markers (Pv02) to 29 (Pv07, excluding LG Pv10). The map had an estimated total genetic length of 1,547.6 cM, with an average distance between molecular markers of 3.10 cM. The sizes of LGs ranged from 89.72 cM (Pv07) to 184.94 cM (Pv08). Two regions with distorted segregation were detected in LGs Pv07 and Pv10. The segregation distortion region in LG Pv07 covered the telomere of chromosome Pv07, which included SNPs in the physical position 41.7 Mb to 51.7Mb. SNPs detected in this region showed a distorted segregation for presence of the BAT93 parental allele. Eight gaps of more than 15 cM were detected across the LGs Pv01, Pv02, Pv03, Pv04, Pv05, and Pv09.

### Genetic analyses of resistance to race 65

**Fig 1:**
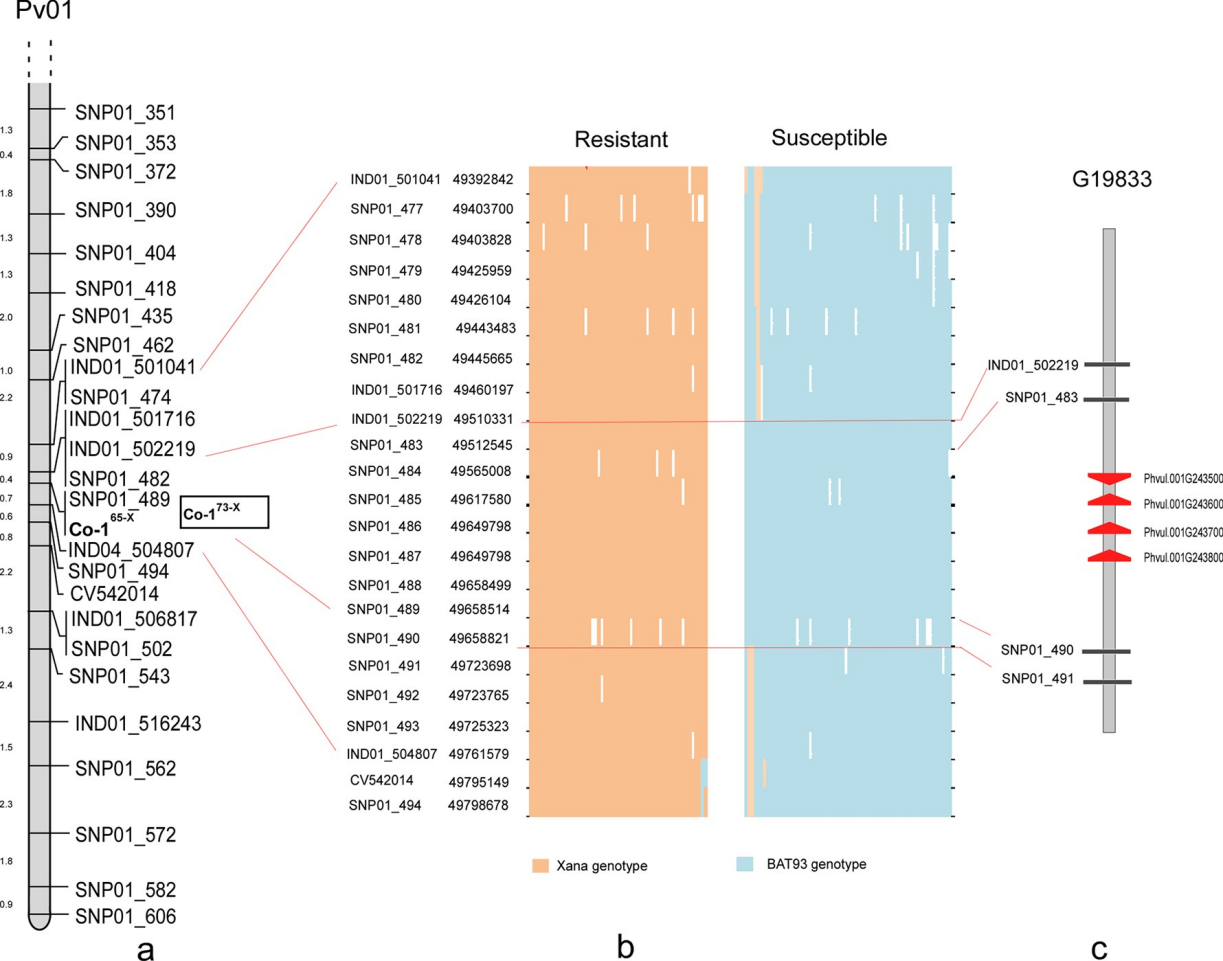
Genetic and physical mapping of the gene conferring resistance to race 65 in the end of linkage group Pv01. A) Fine map obtained for the end of linkage group Pv01 in the XB population. Numbers to the left represent cM distances between markers. B) Graphical representation of genotype observed into the flanked region by SNP01_482 and IND01_504807 based on the physical position of informative (mapped) and redundant SNP markers (non recombination with mapped markers). White gaps represents missing data. C) Genes related with the reaction to biotic stresses annotated in the candidate region for the resistance gene to race 65 (*Co-1*^*65-X*^) in the G19833 genome.

The reactions against race 65 (and remaining races) for the XB RIL populations exhibited discrete segregation; lines with no symtoms (R) or very severe symptoms (S). The observed segregation for resistance to race 65 [Xana(R) × BAT93(S)] fit resistant: susceptible (1R:1S) ratio expected for one resistance gene (Table 1). Linkage analyses between the gene conferring resistance to race 65 and the SNPs included in the linkage map, revealed complete linkage with the molecular markers SNP01_489 [Recombination fraction (RF) = 0.00; LOD = 29.94], located in the distal end of Pv01 (S1 Fig). A fine linkage map was developed around this SNP by adding six new markers, including the molecular marker CV542014 linked to the Co-1 locus (Fig 1A). The resistance gene *Co-1*^*65-X*^ (resistance gene to race 65 locates in the cluster Co-1 in the bean genotype Xana) was mapped between the SNP01_482 (49.44 Mb) and IND01_504807 (49.76 Mb), and no recombinants were detected with the marker SNP01_489 (49.65 Mb) in the resulting map (Fig 1A).

**Table 1:** Observed segregations for resistance to the races 6, 38, 39, 65, 73 and 357 of *C*. *lindemuthianum* in the Xana/BAT93 recombinant inbred population. The segregation ratio considered and the chi-square goodness-of-fit test is indicated in each case. R, resistant; S, susceptible.

	Parental phenotype		XB RIL population						* *
			Observed		Expected				
Race	Xana	Bat93	R	S	R	S	Ratio^a^	χ^2^	*p*
**6**	S	R	57	73	65	65	1: 1	2.11	0.15
**38**	S	R	59	73	66	66	1: 1	1.57	0.21
**39**	S	R	59	63	61	61	1: 1	0.13	0.71
**65**	R	S	57	69	63	63	1: 1	1.20	0.27
**73**	R	R	89	40	97	32	3: 1	2.48	0.12
**357**	S	R	53	74	63.5	63.5	1: 1	3.47	0.06

^a^ Ratio 1 R:1 S corresponds to the expected for one gene in a RIL population; Ratio 3 R: 1 S corresponds to the expected for two independent genes in a RIL population

### Genetic analyses of resistance to races 6, 38, 39 and 357

The observed segregation for resistance to races 6, 38, 39, and 357 [Xana(S) × BAT93(R)] fit the 1R:1S ratio expected for one resistance gene (Table 1). To determine if a single gene or different genes were involved in the response, the co-segregation for resistance to races 6, 38, 39, and 357 was analyzed. Co-segregation among the four races was found in 115 recombinant lines; 62 lines were susceptible to the four races (S^6^S^38^ S^39^S^357^) and 53 lines were resistant (R^6^R^38^R^39^R^357^). Lines XB65 and XB66 showed resistance reactions to race 39, and they were considered recombinant (S^6^S^38^R^39^S^357^), so there are two tightly linked genes: one giving resistance to race 39 and the other resistance to races 6, 38, and 357. Four lines (XB01, XB07, XB218 and XB272) showed a heterozygous response to the resistance. For the remaining lines, seeds or genotypic data were not available.

**Fig 2:**
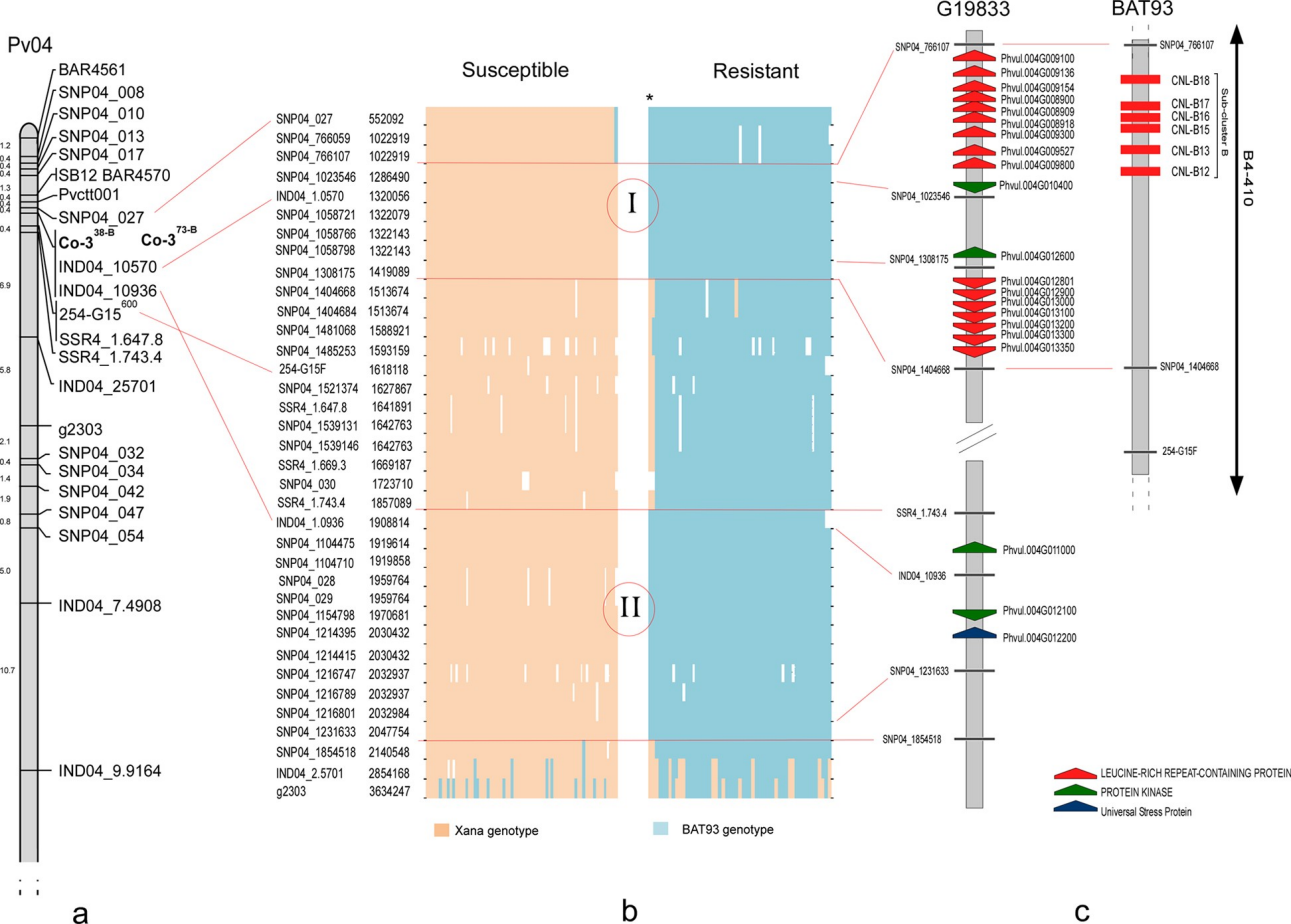
Genetic and physical mapping of the gene conferring resistance to race 38 in the beginning of linkage group Pv04. A) Fine map obtained for the linkage group Pv04 in the region of the cluster Co-3. B) Graphical representation of XB RIL population genotype observed within the region flanked by the markers SNP04_027 and g2303 based on the physical positions of informative (mapped) and redundant SNP markers (non recombination with mapped markers). I and II, regions without recombination in the XB population, all lines showed the same genotype for the loci. White gaps represent missing data. C) Genes related with reaction to biotic stresses annotated in the candidate region for the resistance gene to race 38 (*Co-3*^*38-B*^) in the G19833 genome and in the BAC B4-410 [48]. *, lines XB28 and XB212.

Linkage analyses showed tight linkage between the gene conferring resistance to races 6, 38 and 357 and the molecular marker SNP04_027 (RF = 0.01; LOD = 30.80), located at the beginning of LG Pv04. Due to the low number of SNPs in this region (S1 Fig), 12 additional markers were added to the map: five INDELs, five markers (BAR4561, g2303, 254-G15, Pvct0001, and SB12) reported to be closely linked to *Co* genes in Pv04, and two custom microsatellite markers. The resulting map for the telomeric region contained 19 markers (Fig 2A). The resistance gene *Co-3*^*38-B*^ was mapped between SNP04_027 (0.55 Mb) and 254-G15 (1.61 Mb) & SSR4_1.647.8 (1.64 Mb). Linkage analyses also located the resistance gene to race 39 (*Co-3*^*39-B*^) in the same position, between the markers IND04_10570/ IND04_10936 and 254-G15.

### Genetic analyses of resistance to race 73

Segregation for resistance to race 73 [Xana (R) × BAT93 (R)] fit to the 3:1 R:S ratio expected for two independent genes in RIL populations (Table 1). Contingency chi-squared tests revealed significant associations between the responses to race 73 and two genetic blocks of SNPs in the genetic map (S3 Table): a block between the SNP01_351 and SNP01_582 at the end of LG Pv01, and a second block between the SNP04_008 and SNP04_027 at the beginning of LG Pv04. Contingency chi-squared tests also revealed significant associations between the responses to race 73 and the responses to races 65 (χ^2^ = 58.82; *p* < 0.00001) and 38 (χ^2^ = 32.91; *p* < 0.00001), suggesting the involvement of the Co-1 and Co-3 cluster regions in the resistance to race 73. To confirm this hypothesis, the RIL population was genetically dissected. Two subpopulations, A and B, were established from the original XB RIL population based on the response to race 38 for the former and race 65 for the latter. In subpopulation A, which included 71 lines susceptible to race 38, the segregation observed for responses to race 73 fit the 1:1 ratio (34 R:37 S; χ^2^ = 0.12; *ƿ* = 0.72). No recombinant lines for the responses to races 73 and 65 were detected. The linkage analysis in subpopulation A showed that the resistance to anthracnose race 73 (*Co-1*^*73-X*^) co-segregated with SNP01_489 in the chromosome Pv01 (RF = 0.00; LOD = 21.37) and was flanked by the markers SNP01_482 and INDEL01_504807. The subpopulation B included 64 susceptible lines to race 65. In this population the segregation observed for the resistance to race 73 fit the 1 R:1 S ratio expected for one gene in an RIL population (26 R:36 S; χ^2^ = 1.61; *ƿ* = 0.20). The gene conferring resistance to anthracnose race 73 (*Co-3*^*73-B*^) showed a close linkage with the molecular marker IND04_10570 (RF = 0.01; LOD = 14.82) in chromosome Pv04, and it was flanked by the markers SNP04_027 and 254-G15^600^. A recombinant inbred line for the responses to races 38 and 73 (R^38^ S^73^) was detected, indicating the presence of two closely linked resistance genes. This genetic position corresponds with the position to which the gene *Co-3*^*38-B*^ was mapped. This result suggested that the resistance to race 73 in the XB population was controlled by two independent genes, one from Xana located in chromosome Pv01 (*Co-1*^*73-X*^) and the other from BAT63 in located in chromosome Pv04 (*Co-3*^*73-B*^).

### Annotated genes in within the mapped gene intervals

The linkage analysis revealed that the resistance gene to race 65 (*Co-1*^*65-X*^) was flanked by the markers SNP01_482 and IND01_504807, which corresponded to the physical positions 49,445,665 and 49,761,579 bp in Pv01 (S4 Table). The exploration of genotypes for all available markers (22, including informative and redundant markers) in this genetic region revealed a non-recombinant region located between 49,512,545 (tagged by SNP01_483) and 49,658,821 bp (SNP01_490) (Fig 1B). *In silico* exploration in the G19833 genome revealed a total of 18 genes annotated in this region, four of them serine/threonine-protein kinases (Phvul.001G243500, Phvul.001G243600, Phvul.001G243700, Phvul.001G243800), while the BAT93 genome showed 17 annotated genes (S5 Table).

The linkage analysis indicated that the resistance gene to race 38 (*Co-3*^*38-B*^) was flanked by the markers SNP04_027 and 254-G15, which corresponded to the physical positions 552,092 and 1,618,118 bp, respectively. The exploration of the genotypes for all markers (22 including informative and redundant markers) within and around both flanking markers revealed two non-recombinant regions in which the 54 resistant lines had the BAT93 genotype and the 71 susceptible lines had the Xana genotype for all of the markers: Region I expanded between the positions 1,286,490 (tagged by SNP04_1023546) and 1,419,089 (tagged by SNP04_1308175) and Region II expanded between the positions 1,908,814 (tagged by IND04_10936) and 2,047,754 (tagged by SNP04_1231633) (see Fig 2B). An interposed region, between the positions 1,513,674 (tagged by SNP04_1404668) and 1,857,089 (tagged by SSR4_17434) was detected between both regions. Two resistant lines (XB28 and XB212) showed the Xana genotype for the markers tagging this interposed region, suggesting the BAT93-derived resistance was not located at this position (Fig 2B). Region I contained 9 annotated genes in the G19833 reference genome, including a protein kinase (Phvul.004G012600). Interestingly, on the borders of this region there are two cluster with 9 and 7 leucine-rich repeat-containing protein (see Fig 2). The Region II has 13 annotated genes, one related to biotic stress response (Phvul.004G012200) and a protein kinase (Phvul.004G012100) (see Fig 2).

The makers showing co-segregation with the resistance to race 38 were aligned with the BAT93 genome in two scaffolds of the chromosome Pv04 (S5 Table). These markers tagged 11 genes whose functional annotations were not available [47]. However, three bacterial artificial chromosomes (BACs), BAC B4-410 (FJ817291), BAC 48-B10 (FJ817289), and FZ-E9 (FJ817290), for the telomeric region of chromosome Pv04 were functionally annotated from the bean genotypes BAT93 [48]. Tag sequences with SNP SNP04_ 1404668 and SNP04_766059 flanking *Co-3*^*38-B*^ (Fig 2) aligned to the end of clone BAC B4-410 (at 315,039 and 404,417 bp, respectively), indicating that the resistance gene was located between those positions in B4-410 (Fig 2C). The *R* genes CNL-B12, CNL-B13, CNL-B16, CNL-B17, and CNL-B18, forming the subcluster B were manually annotated in this region (Fig 2C); [48]. The GA-G16 sequence (0 cM to Co-9) also aligns with BAC B4-410 at the positions 343,881 to 344,644 bp (score 1059, identities 92%), which is where our results positioned the *Co-3*^*38-B*^ gene (Fig 2C)

## Discussion

The identification of anthracnose resistance genes have been based on results of allelism tests and/or linkage analyses. Gene characterizations based on a linkage analysis with markers positioned in the genetic map are affected by the variation in recombination frequency among segregating populations, the size and type of segregating population and the occurrence of epistatic or linked genes. Recently, the bean genome became available, which allows for a more detailed characterization of the genes involved in the response to anthracnose. Here, we performed a forward genetic analysis to determine the physical positions in the bean genome of the anthracnose resistance genes detected in the bean genotypes BAT93 and Xana.

**Fig 3:**
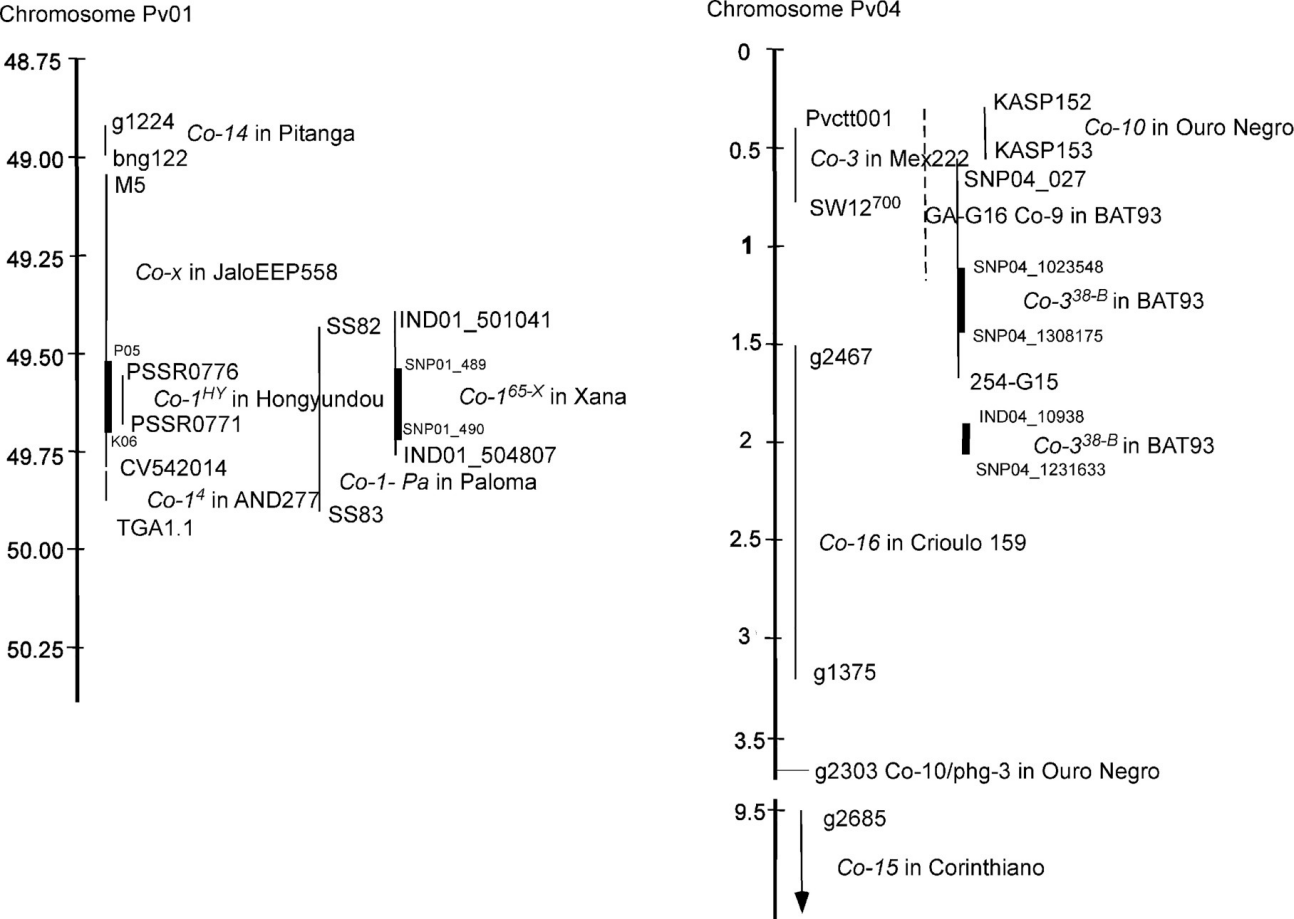
**Representation of physical positions (Mb) deduced from the flanking markers for the Co- genes mapped in the end of bean chromosome Pv01 (left) and in the beginning of the chromosome Pv04 (right).** Physical distance is millions of base pairs (Mb) and alignments were performed using the bean genome V2.1 (www.phytozome.net). Thick bar indicates physical delimited regions from XB RIL map. See S1 Table too. Gene *Co-9* was renamed as *Co-3*^*3*^ and *Co-10* as *Co-3*^*4*^ (http://arsftfbean.uprm.edu/bic/wp-content/uploads/2018/04/Bean_Genes_List_2017.pdf).

The results indicated that the resistance gene to race 65 from Xana (*Co-1*^*65-X*^) was located at the end of Pv01, closely linked to marker CV542014 (2.1 cM), which was physically positioned at 49.79 Mb. This gene was positioned by flanking markers 49.46–49.76 Mb in the bean genome V2.1. This genetic position agrees with the location found in the RIL population Xana × Cornell49242 for the resistance gene to this race [6]. This same CV542014 marker flanks the *Co-1*^*4*^ allele (0.7 cM) in the population AND277 × Ouro Negro [15], and was linked to the *Co-14* gene (4.3 cM) in the population Pitanga × AB136 [22]. The *Co-x* gene from the genotype JaloEEP558 was located in a similar genomic position, flanked by markers M5 and CV542014, which corresponded to the positions 49.09 and 49.60 Mb, respectively [19]. Additionally, the *Co-Pa* gene, described in the cultivar Paloma, was mapped in the F_2:3_ population Paloma × PI207262 [20] on Pv01 between markers SS82 and SS83 located at 49.44 and 49.82 Mb, respectively (see Fig 3). Five genes (Phvul.001G243600, Phvul.001G243700, Phvul.001G243500, Phvul.001G243700, and Phvul.001G244000), located in the delimitated physical region (49,510, 331–49,723,765 bp), were differentially expressed in response to races 81 and 73 in the genotypes Hongyoundou [17] and a near isogenic line derived from the cross Jaguar × Puebla 152, respectively [49]. Also, the kinase Phvul.001G243800 (49,582,832–49,585,847 bp) was significantly associated with responses against races 65, 73, and 3481 in a GWAS involving 230 Andean bean lines [12]. Our results suggest that all of the anthracnose resistance genes mentioned above (*Co-1*^*65-X*^, *Co-1*^*4*^, *Co-14*, *Co-x*, *Co-Pa*, *Co-1*^*HY*^) reported in different bean genotypes, and positioned the distal end of Pv01, are located in a small genomic region, forming part of the same resistance gene-cluster, the Co-1 cluster, which includes four annotated genes with serine/threonine-protein kinase functions (see Fig 3).

Concerning the resistance to race 38, a major resistance gene from BAT93 (*Co-3*^*38-B*^) was located at the beginning of Pv04. Resistance to races 6 and 357 co-segregated with the resistance to race 38, suggesting that the same gene or very closely linked genes control the reaction. The gene *Co-3*^*38-B*^ was located close to markers 254-G15, SB12, g2303, and Pvctt001, linked to race-specific genes mapped in the beginning of Pv04 [9, 26, 28]. The gene *Co-3*^*38-B*^ was flanked by the markers SNP04_027 (552,092 bp) and 254-G15 (1,618,118 bp), even though its position was physically delimited in two separated regions (see Fig 2B); Region I (Pv04:1,286,490..1,419,089 bp) tagged by marker IND04_10570 and Region II (Pv04:1,908,814..2,047,754 bp) tagged by marker IND04_10936. This result is consistent with the observed co-segregation among the resistance and the two markers tagging both regions; IND04_10570 and IND04_10936. This differentiation of the tagged region into two suggests a potential misassembly of the bean genome sequence in this region or a possible rearrangement in one or more of the parental lines, with respect to the standard genome sequence. The beginning of chromosome Pv04 is a complex region containing many *R* gene copies [11], which can cause problems in the assembly, annotation, and sequence alignment owing to the repetitive nature of *R* genes. The identification of two regions nearby may also be the result of the presence of two complementary resistance genes, one in each region, that are both necessary to express the resistance.

Region I included two genes encoding protein kinases (Phvul.004G012600; Phvul.004G010400), and this region is bordered by a cluster of seven genes that encode proteins with LRR domains. Both genes encode typical R proteins involved in responses to biotic stress. R proteins commonly detect the pathogen and activate downstream signaling lending to pathogen resistance [10]. The available data did not allow us to eliminate the involvement of some of the genes located in non-tagged positions adjacent to the physically delimitated regions. In contrast, in the Region II there were no annotated genes that encoded typical R proteins, even though there was a gene encoding a universal stress protein family (Phvul.004G012200). These are small cytoplasmic proteins and their expression levels are affected during prolonged exposure to stress agents, enhancing the rate of cell survival [50]. Additionally, the gene *Co-3*^*38-B*^ was located at the end of BAC B4-410 (positions 315,136–404,480) in subcluster B, which contains six *R* genes and one pseudogene.

On the other hand, results also suggested that at the beginning of chromosome Pv04 there can be differentiated genomic regions containing anthracnose resistance genes, which is consistent with the discontinuous distribution of *R* genes reported by Wu et al [51]. In total, 51 genes encoding LRR proteins were annotated between the positions 0.4 and 2 Mb, with 21 of them grouped in five subclusters of at least 4 R genes. The original *Co-3* gene described in Mexico 222 was mapped using the F_2:3_ population of Mexico 222 × Widusa, flanked by markers Pv-ctt001 and SW12^700^ [9] located at 0.45 and 0.72 Mb, respectively (S1 Table). The original gene *Co-10* from the genotype Ouro Negro was linked to the SCAR marker SF10 [27] that cannot be physically positioned owing to its multiple alignments across chromosome Pv04. The *Co-10* gene was considered an allele of *Co-3* (renamed as *Co-3*^*4*^) and was mapped at 0 cM of the g2303 marker (3.63 Mb) using races 7 and 73 of the pathogen [28]. Later, the *Co-3*^*4*^ allele in Ouro Negro was flanked by the markers KASP152 and KASP153 [29], which had primer sequences that aligned at the 0.43-Mb and 0.51-Mb positions, respectively, on bean chromosome Pv04 (S1 Table). The *Co-15* gene from Corinthiano was linked to marker g2685 (5.8 cM) and mapped between the markers g2685 and g128, which corresponded to positions 9.43 and 32.4 Mb, respectively [33]. The *Co-16* gene from Crioulo 159 was linked to marker g2467 (4.8 cM) and mapped between the markers g2467 and g2303, which corresponded to positions 1.53 and 3.63 Mb, respectively [34]. Finally, the *Co-9* gene (*Co-3*^*3*^) from the genotype BAT93 was closely linked to markers 254-G15F and GA-G16 at 2 and 0 cM, respectively [26]. The 254-G15 marker aligned in position 1.61 Mb, while the GA-G16 sequence aligned in six main positions in the G19833 genome of chromosome Pv04 (between 322,540 and 1,237,351 bp). Thus, considering the physical positions of flanking markers, the results suggest a scenario in which, in the beginning of chromosome Pv04, there are at least two main resistance regions, even though fine mapping would be necessary to locate *Co-15* and *Co-16*: one region contains the Co-3 cluster (0.45 Mb—2.1 Mb considering the physical positions of boundary molecular markers), including *Co-3*, *Co-9*, *Co-10*, Co-*16*, and the resistance genes located in this work (Co-3^38-B^ and Co-3^73-B^), and another region (9.43–32.4 Mb) containing the *Co-15* gene (Fig 3). In this first region, the discontinuous distribution of R genes in the bean genome also agrees with the presence of sub-clusters [51] that may correspond to different genes mapped within the cluster Co-3.

The identification of anthracnose resistance gene based on results of allelism tests is affected by the availability of the specific bean genotypes and the races or isolates used in the identification of each anthracnose resistance gene. In very specific interactions, such as that between *C*. *lindemuthianum* and *P*. *vulgaris*, the genotype or isolate used is an important factor that influences the results of the allelism test. Variation among the identified genes in a bean genotype can be caused by differences in the fungal genotype, even though the isolates were classified as the same race. Differences in pathogenicity have already been observed when different isolates, classified as race 65 using the standardized set of 12 cultivars, were inoculated into other bean genotypes [52]. Also, the interpretation of allelism test results can lead to errors if detailed knowledge regarding the genetics of the genotype used as the reference is lacking. For example, race 73 has been used in many studies investigating the inheritance of anthracnose resistance. The results indicated that BAT93 had a resistance gene to race 73 (*Co-3*^*73-B*^) positioned in the regions at 1.28–1.41 Mb / 1.90–2.04 Mb of chromosome Pv04. Evidence regarding one resistance gene to race 73 in BAT93 corroborated the 120 R:9 S segregation ratio observed in the F_2_ progeny derived from Widusa (R) × BAT93 (R) [16]. However, the gene conferring resistance to race 73 in Widusa was also mapped in the Co-3 cluster (flanking markers PV-cctt001 –SW12) [9]. The 192 R:13 S segregation observed in the response to race 73 of F_2_ segregants derived from Crioulo 159 (R) × BAT93 (R), suggested independence between *Co-16* of Crioulo 159 and *Co-3*^*73-B*^ [34]. No segregation would be expected when the parents share the same resistance locus, but segregation could be observed in the case of two dominant and linked genes in the repulsion phase. In this case, the expected ratios for the F_2_ progeny are: R [1− (1/4r^2^)]: S (1/4r^2^), where r is the recombination fraction. Recombination fractions greater than 0.25 can lead to segregations that fit 15 R:1 S (p > 0.05). Telomeric regions generally show a high rate of recombination [46]. A scenario with two linked genes agrees with the identification of the differentiated physicals regions carrying anthracnose resistance genes in this study.

## Conclusions

A review of the anthracnose resistance genes mapped at the end of LG Pv01 and the beginning of chromosome Pv04 revealed a complex scenario in which many reported genes from different genotypes had similar genetic positions. Fine mapping allowed the positioning of the gene conferring resistance to race 65 in Xana and race 38 in BAT93 in the bean genome, and a comparison of the physical positions of the anthracnose resistance genes previously mapped in both regions. The resistance gene to race 65 in Xana was mapped in the end of LG Pv01 and positioned in the bean genome with other anthracnose resistance genes previously mapped in this genetic position, forming a resistance cluster, cluster Co-1. The resistance gene to race 38 in BAT93 was mapped in the beginning of LG Pv04 and positioned in the bean genome with other anthracnose resistance genes also mapped in this genetic position, forming a wide resistance cluster, cluster Co-3. The discontinuous distribution of R genes in the underlying genomic region suggests the presence of sub-clusters that may correspond to different genes mapped within the cluster Co-3. The results indicated the need for the accurate differentiation of anthracnose resistance genes considering both bean and fungal genotype. The physical positioning of genes in the bean genome should be taken into account when characterizing anthracnose resistance genes located within gene clusters such as Co-1 and Co-3 in the future.

## Supporting information

S1 Fig Genetic linkage maps obtained from Xana/BAT93 RIL population. The linkage map was drawn with the help of the program MapChart 2.32
(PDF)

S1 Table List of markers linked to anthracnose resistance genes mapped in the end of linkage group Pv01 or in the beginning of linkage group Pv04. Physical positions obtained from alignments of the sequences of the specific primers or the amplicons and the G19833 genome of *Phaseolus vulgaris* V2.1 using the BLAST (Basic Local Alignment Search Tool) algorithm are indicated.
(PDF)

S2 Table Linkage map summary information for the Xana/BAT93 recombinant inbred line population including only SNP obtained from Genotyping by sequencing. (PDF)

S3 Table Results of chi-square tests to fit to the expected segregation (1:1) of all the SNPs included in the genetic map. Results of contingency test between the response to race 73 and all the SNPs included in the genetic map. Significant associations were considered after application of Bonferroni correction (α = 0.05).
(PDF)

S4 Table Sequences used in this work. A) Tags sequences including the SNPs used in this work. B) Primer sequences of SSR develop for this work
(PDF)

S5 Table List of annotated genes in the bounded regions obtained from the G19833 genome and BAT93 genome. A/ List of annotated genes found for the bounded region in the chromosome Pv01 from the G19833 genome. Red markers did not show recombination with the resistance locus to race 65 (see Fig 1) b/ List of annotated genes found for the bounded region in the chromosome Pv01 from the BAT93 genome. Red markers did not show recombination with the resistance locus to race 65. c/ List of annotated genes found for the bounded region in the chromosome Pv04 from the G19833 genome. Red markers did not show recombination with the resistance locus to race 38. * Functional annotation related to pathogen resistance. d/ List of annotated genes found for the bounded region in the chromosome Pv04 from the BAT93 genome. Red markers did not show recombination with the resistance locus to race 38.
(PDF)
