# Supplementary material for: Integrating genetic and physical positions of the anthracnose resistance genes described in bean chromosomes Pv01 and Pv04
Source: PLoS One. 2019 Feb 14;14(2):e0212298. doi: 10.1371/journal.pone.0212298 (PMC6375601; doi:10.1371/journal.pone.0212298)
Supplement: S1 Fig — The linkage map was drawn with the help of the program MapChart 2.32 (PDF) [file pone.0212298.s001.pdf]

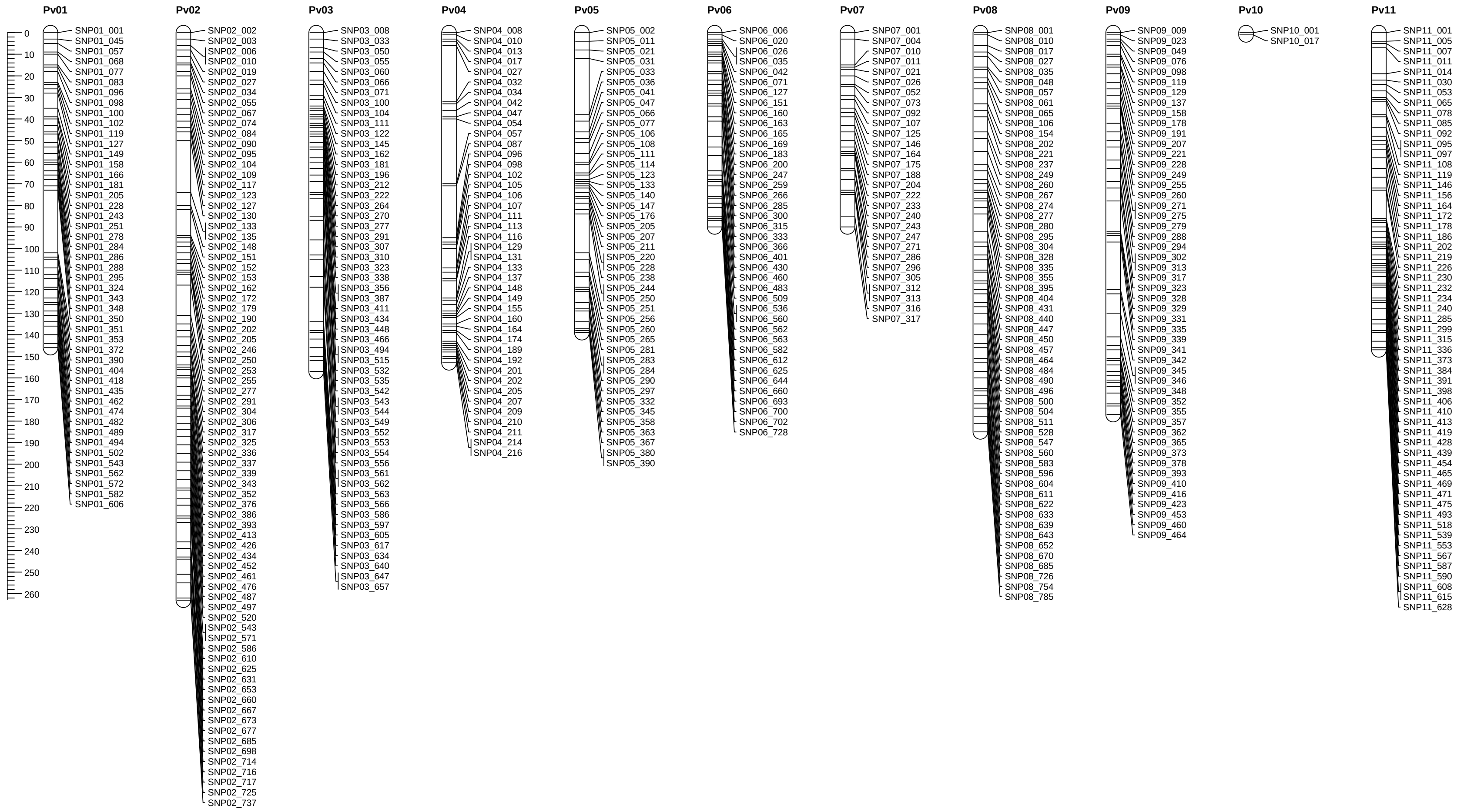

Fig S1. Genetic linkage maps obtained from Xana/BAT93 RIL population. Population was genotyping using SNP markers obtained from genotyping by sequencing. Scale on left of chromosomes shows the genetic distance (cM). The linkage map was drawn with the help of the program MapChart 2.32
