# Supplementary material for: Integrating genetic and physical positions of the anthracnose resistance genes described in bean chromosomes Pv01 and Pv04
Source: PLoS One. 2019 Feb 14;14(2):e0212298. doi: 10.1371/journal.pone.0212298 (PMC6375601; doi:10.1371/journal.pone.0212298)
Supplement: S1 Table — Physical positions obtained from alignments of the sequences of the specific primers or the amplicons and the G19833 genome of Phaseolus vulgaris V2.1 using the BLAST (Basic Local Alignment Search Tool) algorithm are indicated. (PDF) [file pone.0212298.s002.pdf]

S1 Table List of markers linked to anthracnose resistance genes mapped in the end of linkage group Pv01 or in the beginning of linkage group Pv04. Physical positions obtained from alignments of the sequences of the specific primers or the amplicons and the G19833 genome of *Phaseolus vulgaris* V2.1 using the BLAST (Basic Local Alignment Search Tool) algorithm are indicated.

Marker locus	Linked gene	Chromosome	Bean Genotype	cM	*C.lindenmuthianum* race	Polymorphism in XB population	Aligned fragment	Physical Position	Reference
**Pvctt001**	***Co-3***	Pv04	Mexico222	2	19 / 31	Polymorphic	Amplicon	Chr04:455025..459030	[9] Rodriguez-Suarez et al., (2008) Theor Appl Genet 116:807-814
**SW12**	***Co-3***	Pv04	Mexico222	1.1	19 / 31	Monomorphic	Amplicon	Chr04:729048..729808	[9] Rodriguez-Suarez et al., 2008) Theor Appl Genet (116:807-814
**SB12**	***Co-3^3 (Co-9)***	Pv04	A495	2.8	38	Polymorphic	Amplicon	Chr04:289412..289696	Mendez de Vigo et al., (2005) Euphytica 141: 237–245
**254-G15**	***Co-3^3 (Co-9)***	Pv04	BAT93	2	-	Polymorphic	Amplicon	Chr04:1618118..1618904	[26] David et al., (2008) Mol Genet Genomics 280:521-533
**GA-G16R**	***Co-3^3 / Co-y***	Pv04	BAT93	0	-	Monomorphic	Amplicon	Chr04:322540..323590	[26] David et al., (2008) Mol Genet Genomics 280:521-533
**D1174**	***Co-3^3 (Co-9)***	Pv04	BAT93	4	strains 38 / C531 / E29b / E33c	-	-	no available sequence	[18] Geffroy et al., MPMI (1999) 12:774-784
**D1174**	***Co-y / Co-z***	Pv04	JaloEEP531	4	strains M38 / A47 / 88 / 80	-	-	no available sequence	[18] Geffroy et al., MPMI (1999) 12:774-784
**g2303**	***Co-3^4***	Pv04	Ouro Negro	0	73 / 7	Polymorphic	Amplicon	Chr04:3634247..3634637	[28] Gonçalves-Vidigal et al., (2013) Theor App Genet 126:2245-2255
**BARCPVSSR4561**	***Co-3^4 (Co-10)***	Pv04	Ouro Negro	0	73	Polymorphic	Primer F/ R	Chr04:59622..59641	Valentini et al., (2015) Ann Rep Bean Improv Coop 58:21-22
**BARCPVSSR4570**	***Co-3^4 (Co-10)***	Pv04	Ouro Negro	0	73	Polymorphic	Primer F/ R	Chr04:136413..136434	Valentini et al., (2015) Ann Rep Bean Improv Coop 58:21-22
**KAPS153**	***Co-3^4 (Co-10)***	Pv04	Ouro Negro	0.1	73	-	Primer F/ R	Chr04:516291..516322	[29] Valentini et al., (2017) Thero Appl Genet 130(8):1705-1722
**KASP152**	***Co-3^4 (Co-10)***	Pv04	Ouro Negro	0.3	73	-	Primer F/ R	Chr04:430278..430311	[29] Valentini et al., (2017) Thero Appl Genet 130(8):1705-1722
**SCF10**	***Co-3^4 (Co-10)***	Pv04	Ouro Negro	7.8	73 / 7	Monomorphic	Amplicon	Chr04:10060047..10060237	[28] Gonçalves-Vidigal et al., (2013) Theor App Genet 126:2245-2255
**g128**	***Co-15***	Pv04	Corinthiano	-	2047	Monomorphic	Amplicon	Chr04:32414092..32414596	[33] Sousa et al., Crop Sci (2015) 55:1900-1910

**g2685**	***Co-15***	Pv04	Corinthiano	5.6	2047	Monomorphic	Amplicon	Chr04:9432203..9432817	[33] Sousa et al., Crop Sci (2015) 55:1900-1910
**g1375**	***Co-16***	Pv04	Crioulo 159	-	73 / 2047	Monomorphic	Amplicon	Chr04:3197283..3197690	[34] Coimbra-Gonçalves et al., (2016) Crop Sci 56:1-12
**g2467**	***Co-16***	Pv04	Crioulo 159	4.8	73 / 2047	Monomorphic	Amplicon	Chr04:1537301..1537802	[34] Coimbra-Gonçalves et al., (2016) Crop Sci 56:1-12
**$OF10_{1072}$**	***$Co\text{-}3^4$ (Co-10)***	Pv04	Ouro Negro	6.0	-	Polymorphic	Primer F/ R	No aligment	Corrêa et al., Crop Sci (2000) 40:804-807
**CV542014**	***$Co\text{-}1^4$***	Pv01	AND277	0.7	65 / 73 /2047	Polymorphic	Amplicon	Chr01:49795149..49795765	[15] Gonçalves-Vidigal et al., Theor Appl Genet (2011) 122:893-903
**TGA1.1**	***$Co\text{-}1^4$***	Pv01	AND277	1.3	65 / 73 /2047	Monomorphic	Primer F/ R	Chr01:49795615..49795639	[15] Gonçalves-Vidigal et al., Theor Appl Genet (2011) 122:893-903
**M5**	***$Co\text{-}1^4$***	Pv01	JaloEEP558	3.3	strain 100	-	Primer F/ R	Chr01:49092455..490924749	[19] Richard et al., (2014) Theor Appl Genet 127:1653
**CV542014**	***Co-x***	Pv01	JaloEEP558	2.7	strain 100	Polymorphic	Amplicon	Chr01:49795149..49795765	[19] Richard et al., (2014) Theor Appl Genet 127:1653
**IND01_502219**	***Co-1***	Pv01	Jaguar	-	73	Polymorphic	Amplicon	Chr01:49510331..49510484	[12] Zuiderveen et al., (2016 PLoS ONE) 11(6): e0156391
**SS82**	***Co-1-Pa***	Pv01	Paloma	1.3	2047 /3481	-	Primer F/ R	Chr01:49444377..49444405	[20] Castro et al (2017) BMC Genomic 18:306
**SS83**	***Co-1-Pa***	Pv01	Paloma	2.1	2047 /3481	-	Primer F/ R	Chr01:49828401..49828427	[20] Castro et al (2017) BMC Genomic 18:306
**PSSR0776**	***$Co\text{-}1^{HY}$***	Pv01	Hongyundou	0.7	81	-	Primer F/ R	Chr01:49515883..49515902	[17] Chen et al (2017) PLoS ONE 12(1): e0169954
**PSSR0771**	***$Co\text{-}1^{HY}$***	Pv01	Hongyundou	0.2	81	-	Primer F/ R	Chr01:49621098..49621117	[17] PLoS ONE (2017) 12(1): e0169954
**g1224**	***Co-14***	Pv01	Pitanga	1.3	2047	-	Amplicon	Chr01:48999062..48999518	Gonzalvez-Vidigal et al 2016 Ann Rep Bean Improv Coop 59: 55-56
**bng122**	***Co-14***	Pv01	Pitanga	1.7	2047	-	Amplicon	Chr01:49006976..49007522	Gonzalvez-Vidigal et al 2016 Ann Rep Bean Improv Coop Ann Rep Bean Improv Coop 59: 55-56

Additional references

Correa, R.X., M.R. Costa, P.I. Good-God, V.A. Ragagnin, F.G.Faleiro, M.A. Moreira & E.G. Barros, 2000. Sequence characterized amplified regions linked to rust resistance genes in the common bean. Crop Sci 40: 804–807.

Gonçalves-Vidigal MC, Pacheco CMNA, Vidigal Filho PS, Lacanallo GF, Sousa LL, Martins VSR.2016 Genetic mapping of the anthracnose resistance gene co-14 in the common bean cultivar PITANGA Ann Rep Bean Improv Coop 59: 55-56

Mendez-Vigo, B., Rodr´ıguez-Suarez, C., Pa˜neda, A., Ferreira,J.J., Giraldez, R. 2005. Molecular markers and allelic relationships of anthracnose resistance gene cluster B4 in common bean. Euphytica 141:237–245.

Valentini G., M.C. Gonçalves-Vidigal, P.B. Cregan, Q. Song, M.A. Pastor-Corrales (2015) Cluster with the ur-14 rust resistance gene Ann Rep Bean Improv Coop 58:21-22
