# Supplementary material for: Integrating genetic and physical positions of the anthracnose resistance genes described in bean chromosomes Pv01 and Pv04
Source: PLoS One. 2019 Feb 14;14(2):e0212298. doi: 10.1371/journal.pone.0212298 (PMC6375601; doi:10.1371/journal.pone.0212298)
Supplement: S2 Table — (PDF) [file pone.0212298.s003.pdf]

S2 Table. Linkage map summary information for the Xana/BAT93 recombinant inbred line population including only SNP obtained from Genotyping by Sequencing.

Linkage Groups	SNP	Non distorted segregation (p>0.05)	Distorted segregation (p<0.05)	Informative SNP	Total distance (cM)	Average distance (cM)	Average distance (bp)
**Pv01**	826	610	216	47	145.54	3.09	1106646.87
**Pv02**	825	738	87	76	263.00	3.44	644271.82
**Pv03**	761	661	100	55	157.24	2.85	948608.55
**Pv04**	277	216	61	42	153.22	3.64	1087858.24
**Pv05**	460	393	67	43	138.79	3.22	940068.12
**Pv06**	738	734	4	40	90.11	2.25	798134.93
**Pv07**	645	330	315	29	89.72	3.09	1385179.00
**Pv08**	800	793	7	56	184.94	3.30	1061830.00
**Pv09**	796	468	328	50	176.72	3.53	740789.52
**Pv10**	386	17	369	2	1.11		
**Pv11**	631	630	1	57	147.27	2.58	879197.28
	7145	5590	1555	497	1547.6	3.10	959258.43
