# Supplementary material for: Integrating genetic and physical positions of the anthracnose resistance genes described in bean chromosomes Pv01 and Pv04
Source: PLoS One. 2019 Feb 14;14(2):e0212298. doi: 10.1371/journal.pone.0212298 (PMC6375601; doi:10.1371/journal.pone.0212298)
Supplement: S3 Table — Results of contingency test between the response to race 73 and all the SNPs included in the genetic map. Significant associations were considered after application of Bonferroni correction (α = 0.05). (PDF) [file pone.0212298.s004.pdf]

Table S3. **Results of chi-square tests to fit to the expected segregation (1:1) of all the SNPs included in the genetic map.** Results of contingency test between the response to race 73 and all the SNPs included in the genetic map. Significant associations were considered after application of Bonferroni correction (α = 0.05).

Name	**LG**	Genetic position	chi-square (1:1)	*P*	Chi cont R73	*p*
SNP01_001	**Pv01**	0	0.2609	0.6095	2.59	0.107729739
SNP01_045	**Pv01**	3.25	0.4923	0.4829	2.06	0.151285565
SNP01_057	**Pv01**	5.41	0.8963	0.3438	1.97	0.160605142
SNP01_068	**Pv01**	8.73	0.8963	0.3438	0.66	0.417946868
SNP01_077	**Pv01**	10.43	0.1852	0.6670	0.32	0.572701084
SNP01_083	**Pv01**	14.69	0.0294	0.8638	0.83	0.363149164
SNP01_096	**Pv01**	16.3	0.0667	0.7963	0.61	0.435910844
SNP01_098	**Pv01**	17.82	0.0074	0.9314	0.35	0.553165365
SNP01_100	**Pv01**	22.54	0.2647	0.6069	0.00	0.968958255
SNP01_102	**Pv01**	24.1	0.4706	0.4927	0.25	0.618304466
SNP01_119	**Pv01**	26.41	0.5912	0.4419	0.17	0.682205573
SNP01_127	**Pv01**	28.07	0.7463	0.3877	0.01	0.905535899
SNP01_149	**Pv01**	35.44	0.4706	0.4927	0.31	0.578794762
SNP01_158	**Pv01**	39.42	0.8832	0.3473	0.03	0.871051182
SNP01_166	**Pv01**	40.21	0.7463	0.3877	0.13	0.720962595
SNP01_181	**Pv01**	43.13	0.8832	0.3473	0.01	0.916458534
SNP01_205	**Pv01**	45.8	1.2901	0.2560	0.41	0.522558785
SNP01_228	**Pv01**	46.63	0.2687	0.6042	0.18	0.672047118
SNP01_243	**Pv01**	50.71	0.0074	0.9314	0.61	0.435910844
SNP01_251	**Pv01**	53.15	0.0677	0.7948	0.00	0.950853021
SNP01_278	**Pv01**	56.4	0.2647	0.6069	0.03	0.866399372
SNP01_284	**Pv01**	58.84	0.8832	0.3473	0.08	0.774814981
SNP01_286	**Pv01**	59.65	2.1407	0.1434	0.00	0.997291590
SNP01_288	**Pv01**	60.67	2.3478	0.1255	0.01	0.927169622
SNP01_295	**Pv01**	63.8	1.2519	0.2632	0.13	0.722578291
SNP01_324	**Pv01**	66.13	2.0496	0.1522	0.35	0.553059063
SNP01_343	**Pv01**	68.48	3.5072	0.0611	0.35	0.556277732
SNP01_348	**Pv01**	70.55	3.5588	0.0592	0.24	0.626187174
SNP01_350	**Pv01**	73.47	3.9185	0.0478	1.16	0.280830618
**SNP01_351**	**Pv01**	**102.27**	**3.7518**	**0.0528**	**15.56**	**0.000079958**
**SNP01_353**	**Pv01**	**104.28**	**2.8169**	**0.0933**	**20.27**	**0.000006732**
**SNP01_372**	**Pv01**	**105**	**1.4412**	**0.2299**	**20.34**	**0.000006488**
**SNP01_390**	**Pv01**	**109.29**	**2.2817**	**0.1309**	**25.93**	**0.000000353**
**SNP01_404**	**Pv01**	**112.13**	**2.5245**	**0.1121**	**32.02**	**0.000000015**
**SNP01_418**	**Pv01**	**114.32**	**2.9851**	**0.0840**	**27.93**	**0.000000126**
**SNP01_435**	**Pv01**	**117.72**	**0.3475**	**0.5555**	**36.36**	**0.000000002**
**SNP01_462**	**Pv01**	**119.41**	**0.0000**	**1.0000**	**34.67**	**0.000000004**
**SNP01_474**	**Pv01**	**123.06**	**0.0071**	**0.9329**	**47.50**	**0.000000000**
**SNP01_482**	**Pv01**	**125.24**	**0.2535**	**0.6146**	**52.89**	**0.000000000**

**SNP01_489**	**Pv01**	**126.14**	**0.3475**	**0.5555**	**57.89**	**0.000000000**
**SNP01_494**	**Pv01**	**128.51**	**0.2535**	**0.6146**	**54.69**	**0.000000000**
**SNP01_502**	**Pv01**	**131.21**	**0.5745**	**0.4485**	**45.82**	**0.000000000**
**SNP01_543**	**Pv01**	**133.63**	**0.5745**	**0.4485**	**42.66**	**0.000000000**
**SNP01_562**	**Pv01**	**136.46**	**0.2571**	**0.6121**	**32.27**	**0.000000013**
**SNP01_572**	**Pv01**	**140.45**	**0.0071**	**0.9329**	**20.04**	**0.000007591**
**SNP01_582**	**Pv01**	**144.06**	**0.3525**	**0.5527**	**16.06**	**0.000061496**
SNP01_606	**Pv01**	145.64	0.4923	0.4829	11.12	0.000856323
SNP02_002	**Pv02**	0	3.3158	0.0686	0.04	0.850422230
SNP02_003	**Pv02**	2.95	3.1727	0.0749	0.00	0.953963154
SNP02_006	**Pv02**	5.54	1.6187	0.2033	0.06	0.805146599
SNP02_010	**Pv02**	6.43	2.2817	0.1309	0.07	0.787843499
SNP02_019	**Pv02**	8.11	1.6187	0.2033	0.01	0.924105981
SNP02_027	**Pv02**	10.65	0.8705	0.3508	0.00	0.954611893
SNP02_034	**Pv02**	13.77	0.8832	0.3473	0.01	0.934513559
SNP02_055	**Pv02**	15.43	1.2158	0.2702	0.09	0.765328800
SNP02_067	**Pv02**	17.92	2.5971	0.1071	1.13	0.287700062
SNP02_074	**Pv02**	20.26	2.8571	0.0910	0.30	0.581160083
SNP02_084	**Pv02**	25.83	1.6917	0.1934	0.43	0.510142309
SNP02_090	**Pv02**	28.42	1.8551	0.1732	0.79	0.374105082
SNP02_095	**Pv02**	30.6	3.5072	0.0611	0.95	0.329555001
SNP02_104	**Pv02**	34.69	2.9851	0.0840	2.58	0.108377883
SNP02_109	**Pv02**	38.09	3.2190	0.0728	0.97	0.324720832
SNP02_117	**Pv02**	41.01	3.1727	0.0749	0.40	0.529675169
SNP02_123	**Pv02**	43.93	3.0303	0.0817	0.12	0.728593239
SNP02_127	**Pv02**	45.65	3.8613	0.0494	0.06	0.799284324
SNP02_130	**Pv02**	50.46	3.3664	0.0665	0.03	0.867489553
SNP02_133	**Pv02**	73.75	3.8058	0.0511	1.30	0.253985365
SNP02_135	**Pv02**	74.49	2.3143	0.1282	0.42	0.519050745
SNP02_148	**Pv02**	79.81	2.0791	0.1493	0.54	0.462154960
SNP02_151	**Pv02**	82.19	3.8058	0.0511	1.82	0.176997037
SNP02_152	**Pv02**	93.9	2.1095	0.1464	0.79	0.372933768
SNP02_153	**Pv02**	94.78	1.8551	0.1732	1.52	0.216988369
SNP02_162	**Pv02**	96.75	0.8705	0.3508	0.97	0.325712087
SNP02_172	**Pv02**	98.73	0.6000	0.4386	0.05	0.815032093
SNP02_179	**Pv02**	102.28	0.1159	0.7335	0.31	0.578794762
SNP02_190	**Pv02**	104.57	0.0076	0.9304	0.99	0.320450112
SNP02_202	**Pv02**	106.97	0.1143	0.7353	1.33	0.249059521
SNP02_205	**Pv02**	109.89	0.7143	0.3980	2.07	0.150461701
SNP02_246	**Pv02**	110.62	0.2571	0.6121	2.00	0.157628277
SNP02_250	**Pv02**	112.08	0.5827	0.4452	1.51	0.219494500
SNP02_253	**Pv02**	116.63	2.5971	0.1071	0.90	0.341842757
SNP02_255	**Pv02**	131.26	1.1077	0.2926	3.10	0.078158464
SNP02_277	**Pv02**	135.25	0.0000	1.0000	0.32	0.570908499
SNP02_291	**Pv02**	138.07	0.0657	0.7977	0.59	0.443496530
SNP02_304	**Pv02**	141.21	0.1852	0.6670	0.00	0.945684669
SNP02_306	**Pv02**	145.05	0.0647	0.7991	0.02	0.879598221
SNP02_317	**Pv02**	147.57	0.1176	0.7316	0.14	0.706692140
SNP02_325	**Pv02**	149.98	0.0072	0.9324	0.02	0.894720671
SNP02_336	**Pv02**	153.68	0.5912	0.4419	0.08	0.771427724
SNP02_337	**Pv02**	154.61	0.7353	0.3912	0.43	0.513406394

SNP02_339	**Pv02**	156.27	0.9237	0.3365	1.95	0.163082685
SNP02_343	**Pv02**	159.1	0.2609	0.6095	0.05	0.825649202
SNP02_352	**Pv02**	159.89	0.3630	0.5469	0.25	0.619305860
SNP02_376	**Pv02**	163.9	1.4627	0.2265	0.02	0.899191371
SNP02_386	**Pv02**	167.91	0.2687	0.6042	0.57	0.450053691
SNP02_393	**Pv02**	169.56	1.1077	0.2926	0.04	0.842955054
SNP02_413	**Pv02**	172.73	0.0677	0.7948	0.01	0.904807238
SNP02_426	**Pv02**	174.24	0.0303	0.8618	0.00	0.960678640
SNP02_434	**Pv02**	178.2	0.0076	0.9304	0.03	0.868694197
SNP02_452	**Pv02**	181.37	0.0299	0.8628	0.10	0.746764671
SNP02_461	**Pv02**	183.73	0.0667	0.7963	0.01	0.941010525
SNP02_476	**Pv02**	187.1	0.9098	0.3402	0.08	0.780564045
SNP02_487	**Pv02**	191.32	1.4627	0.2265	0.00	0.945684669
SNP02_497	**Pv02**	195.33	0.5912	0.4419	0.05	0.819949134
SNP02_520	**Pv02**	198.98	0.1852	0.6670	0.29	0.592932533
SNP02_543	**Pv02**	202.56	0.3577	0.5498	0.05	0.819949134
SNP02_571	**Pv02**	203.31	0.0657	0.7977	0.22	0.637505242
SNP02_586	**Pv02**	207.48	0.0000	1.0000	0.05	0.831015163
SNP02_610	**Pv02**	210.8	0.8705	0.3508	0.13	0.714510775
SNP02_625	**Pv02**	212.29	1.2336	0.2667	0.47	0.492209006
SNP02_631	**Pv02**	216.02	3.1727	0.0749	0.97	0.325712087
SNP02_653	**Pv02**	218.93	2.8169	0.0933	0.51	0.474237746
SNP02_660	**Pv02**	223.78	2.3143	0.1282	1.56	0.211219442
SNP02_667	**Pv02**	225.37	1.1986	0.2736	1.73	0.188058102
SNP02_673	**Pv02**	226.8	1.0286	0.3105	0.48	0.489368808
SNP02_677	**Pv02**	236.49	0.5745	0.4485	0.03	0.863617802
SNP02_685	**Pv02**	239.42	0.0303	0.8618	0.00	0.949805226
SNP02_698	**Pv02**	242.94	0.0000	1.0000	0.41	0.520378716
SNP02_714	**Pv02**	243.8	0.0070	0.9334	0.49	0.485491802
SNP02_716	**Pv02**	251.14	0.0286	0.8658	0.14	0.705285628
SNP02_717	**Pv02**	255.28	0.5586	0.4548	0.36	0.550012716
SNP02_725	**Pv02**	261.56	0.0282	0.8667	0.24	0.620876838
SNP02_737	**Pv02**	263	0.0000	1.0000	0.33	0.564312538
SNP03_008	**Pv03**	0	1.4848	0.2230	3.03	0.081924595
SNP03_033	**Pv03**	2.57	0.8963	0.3438	5.01	0.025263659
SNP03_050	**Pv03**	6.69	0.5912	0.4419	4.84	0.027883997
SNP03_055	**Pv03**	8.88	1.0435	0.3070	3.88	0.048849940
SNP03_060	**Pv03**	11.78	0.7246	0.3946	3.75	0.052712196
SNP03_066	**Pv03**	13.95	0.2647	0.6069	6.32	0.011915938
SNP03_071	**Pv03**	17.87	0.0073	0.9319	3.76	0.052462689
SNP03_100	**Pv03**	21.77	0.2571	0.6121	3.23	0.072279187
SNP03_104	**Pv03**	23.5	0.5827	0.4452	2.22	0.136476760
SNP03_111	**Pv03**	28.53	0.0647	0.7991	2.01	0.156597714
SNP03_122	**Pv03**	30.76	0.0000	1.0000	2.82	0.093244473
SNP03_145	**Pv03**	33.73	0.0647	0.7991	1.48	0.223410430
SNP03_162	**Pv03**	35.2	0.0072	0.9324	1.49	0.222623317
SNP03_181	**Pv03**	35.95	0.0294	0.8638	1.68	0.194405130
SNP03_196	**Pv03**	38.08	0.0299	0.8628	2.85	0.091296674
SNP03_212	**Pv03**	38.8	0.0290	0.8648	2.37	0.123945642
SNP03_222	**Pv03**	39.52	0.1159	0.7335	1.17	0.279599167
SNP03_264	**Pv03**	41.91	0.8705	0.3508	3.35	0.067199132

SNP03_270	**Pv03**	43.43	2.2061	0.1375	2.26	0.133017012
SNP03_277	**Pv03**	44.44	1.6187	0.2033	1.90	0.168316654
SNP03_291	**Pv03**	46.47	1.1077	0.2926	1.06	0.303840122
SNP03_307	**Pv03**	47.23	1.2519	0.2632	0.65	0.421111007
SNP03_310	**Pv03**	47.98	1.4000	0.2367	0.55	0.459863951
SNP03_323	**Pv03**	50.82	2.5971	0.1071	0.20	0.651107858
SNP03_338	**Pv03**	52.98	1.8286	0.1763	0.37	0.545511631
SNP03_356	**Pv03**	53.7	1.4203	0.2334	0.15	0.696463437
SNP03_387	**Pv03**	54.48	1.4000	0.2367	0.12	0.725779282
SNP03_411	**Pv03**	58.4	0.0073	0.9319	0.00	0.946473467
SNP03_434	**Pv03**	60.09	0.2571	0.6121	0.01	0.924393521
SNP03_448	**Pv03**	62.64	0.1825	0.6692	0.03	0.853548404
SNP03_466	**Pv03**	65.71	0.4848	0.4862	0.01	0.904807238
SNP03_494	**Pv03**	68.62	0.3577	0.5498	0.15	0.694700673
SNP03_515	**Pv03**	69.43	1.2519	0.2632	0.14	0.708905309
SNP03_532	**Pv03**	73.9	0.4571	0.4990	0.10	0.752681953
SNP03_535	**Pv03**	74.64	1.0286	0.3105	0.09	0.758819229
SNP03_542	**Pv03**	76.86	1.4000	0.2367	0.19	0.667068027
SNP03_543	**Pv03**	84.83	1.8286	0.1763	0.02	0.880417890
SNP03_544	**Pv03**	84.93	1.8286	0.1763	0.02	0.880417890
SNP03_549	**Pv03**	87.19	2.9412	0.0863	0.07	0.793274378
SNP03_552	**Pv03**	96.37	3.2190	0.0728	0.59	0.444047541
SNP03_553	**Pv03**	96.47	2.5971	0.1071	0.83	0.362233090
SNP03_554	**Pv03**	103.14	3.5072	0.0611	1.85	0.173655168
SNP03_556	**Pv03**	105.41	3.1727	0.0749	0.97	0.324432437
SNP03_561	**Pv03**	112.73	3.7518	0.0528	0.80	0.369911914
SNP03_562	**Pv03**	113.42	3.2667	0.0707	2.30	0.129786527
SNP03_563	**Pv03**	118.38	3.7518	0.0528	1.46	0.226385674
SNP03_566	**Pv03**	134.17	2.8986	0.0887	2.11	0.146546251
SNP03_586	**Pv03**	137.52	2.8986	0.0887	0.92	0.336565882
SNP03_597	**Pv03**	139.18	1.4203	0.2334	1.92	0.166266486
SNP03_605	**Pv03**	142.31	0.1212	0.7277	0.63	0.427608431
SNP03_617	**Pv03**	145.93	0.0000	1.0000	1.23	0.266739563
SNP03_634	**Pv03**	150.1	0.1852	0.6670	0.34	0.562507959
SNP03_640	**Pv03**	152.39	0.0000	1.0000	0.07	0.798557650
SNP03_647	**Pv03**	156.6	0.1799	0.6715	0.00	0.944172907
SNP03_657	**Pv03**	157.24	1.8824	0.1701	0.10	0.753350567
**SNP04_008**	Pv04	**0**	**0.8582**	**0.3543**	**19.20**	**0.000011746**
**SNP04_010**	Pv04	**0.95**	**1.5957**	**0.2065**	**20.58**	**0.000005726**
**SNP04_013**	Pv04	**2.82**	**2.5603**	**0.1096**	**25.27**	**0.000000500**
**SNP04_017**	Pv04	**3.54**	**3.4571**	**0.0630**	**28.73**	**0.000000083**
**SNP04_027**	Pv04	**5.75**	**3.8058**	**0.0511**	**36.34**	**0.000000002**
SNP04_032	**Pv04**	32.49	1.5077	0.2195	12.75	0.000356899
SNP04_034	**Pv04**	33.26	4.0000	0.0455	10.30	0.001333005
SNP04_042	**Pv04**	35.62	3.1277	0.0770	9.49	0.002071378
SNP04_047	**Pv04**	38.86	3.0839	0.0791	6.81	0.009041444
SNP04_054	**Pv04**	40.27	3.4085	0.0649	6.50	0.010790636
SNP04_057	**Pv04**	69.79	1.8551	0.1732	1.23	0.267151672
SNP04_087	**Pv04**	70.54	2.6350	0.1045	1.19	0.275865347
SNP04_096	**Pv04**	95.42	1.0286	0.3105	8.75	0.003089966
SNP04_098	**Pv04**	96.87	0.3577	0.5498	10.05	0.001521650

SNP04_102	**Pv04**	98.37	1.1077	0.2926	10.39	0.001263823
SNP04_105	**Pv04**	99.9	0.3577	0.5498	10.59	0.001137171
SNP04_106	**Pv04**	109.45	1.0588	0.3035	4.69	0.030394669
SNP04_107	**Pv04**	111.02	1.0435	0.3070	7.39	0.006545447
SNP04_111	**Pv04**	113.96	0.0000	1.0000	9.58	0.001964400
SNP04_113	**Pv04**	114.7	0.0000	1.0000	10.98	0.000920989
SNP04_116	**Pv04**	123.01	0.2500	0.6171	9.57	0.001975670
SNP04_129	**Pv04**	123.7	0.0282	0.8667	10.57	0.001148145
SNP04_131	**Pv04**	124.39	0.0638	0.8005	11.35	0.000753000
SNP04_133	**Pv04**	126.47	0.0073	0.9319	11.38	0.000743322
SNP04_137	**Pv04**	129.06	0.1159	0.7335	14.31	0.000155410
SNP04_148	**Pv04**	129.93	1.0435	0.3070	13.77	0.000206208
SNP04_149	**Pv04**	130.91	0.0303	0.8618	11.03	0.000897335
SNP04_155	**Pv04**	132.07	0.0638	0.8005	12.86	0.000335902
SNP04_160	**Pv04**	135.33	0.1143	0.7353	10.20	0.001400770
SNP04_164	**Pv04**	136.02	0.0286	0.8658	10.95	0.000934071
SNP04_174	**Pv04**	137.96	0.0638	0.8005	6.91	0.008550403
SNP04_189	**Pv04**	139.17	0.0638	0.8005	6.36	0.011662631
SNP04_192	**Pv04**	142.71	0.2535	0.6146	7.87	0.005039914
SNP04_201	**Pv04**	144.2	0.1773	0.6737	7.30	0.006900200
SNP04_202	**Pv04**	144.89	0.0638	0.8005	7.94	0.004834445
SNP04_205	**Pv04**	145.57	0.0286	0.8658	6.20	0.012788732
SNP04_207	**Pv04**	147.07	0.0282	0.8667	6.48	0.010906847
SNP04_209	**Pv04**	147.78	0.0286	0.8658	6.20	0.012788732
SNP04_210	**Pv04**	150.33	0.2647	0.6069	3.02	0.082000747
SNP04_211	**Pv04**	151.05	0.0075	0.9309	3.53	0.060431287
SNP04_214	**Pv04**	152.68	0.0638	0.8005	4.16	0.041324626
SNP04_216	**Pv04**	153.22	0.4638	0.4959	4.16	0.041336903
SNP05_002	**Pv05**	0	0.1143	0.7353	0.87	0.351751191
SNP05_011	**Pv05**	3.58	0.0282	0.8667	1.36	0.242864436
SNP05_021	**Pv05**	7.89	0.4571	0.4990	3.44	0.063694556
SNP05_031	**Pv05**	11.5	1.3803	0.2401	2.44	0.117941045
SNP05_033	**Pv05**	37.63	2.8571	0.0910	3.45	0.063078359
SNP05_036	**Pv05**	41.3	3.6993	0.0544	3.12	0.077336349
SNP05_041	**Pv05**	45.86	3.9774	0.0461	0.84	0.358663637
SNP05_047	**Pv05**	48.57	3.4571	0.0630	0.76	0.383713618
SNP05_066	**Pv05**	50.75	3.4085	0.0649	0.88	0.347873928
SNP05_077	**Pv05**	55.75	2.0496	0.1522	0.71	0.400878465
SNP05_106	**Pv05**	59.91	1.6187	0.2033	0.44	0.508125336
SNP05_108	**Pv05**	60.78	2.8169	0.0933	1.50	0.220774072
SNP05_111	**Pv05**	64.96	1.0141	0.3139	0.82	0.365521622
SNP05_114	**Pv05**	65.8	0.7692	0.3805	1.17	0.278475437
SNP05_123	**Pv05**	67.95	0.3427	0.5583	1.30	0.253670752
SNP05_133	**Pv05**	69.38	0.1773	0.6737	0.82	0.364270802
SNP05_140	**Pv05**	70.17	0.7143	0.3980	0.61	0.434347028
SNP05_147	**Pv05**	70.95	1.1986	0.2736	0.60	0.439017923
SNP05_176	**Pv05**	71.74	1.0141	0.3139	0.71	0.400878465
SNP05_205	**Pv05**	73.71	1.0141	0.3139	0.82	0.365521622
SNP05_207	**Pv05**	75.68	0.7042	0.4014	0.83	0.363415527
SNP05_211	**Pv05**	77.25	0.7692	0.3805	0.11	0.744707111
SNP05_220	**Pv05**	78.69	0.3525	0.5527	0.72	0.397415123

SNP05_228	**Pv05**	79.4	1.1818	0.2770	1.96	0.161606975
SNP05_238	**Pv05**	82.17	1.1818	0.2770	1.96	0.161606975
SNP05_244	**Pv05**	83.55	2.2500	0.1336	1.64	0.200164692
SNP05_250	**Pv05**	84.27	1.5957	0.2065	2.09	0.147911142
SNP05_251	**Pv05**	102.38	0.0000	1.0000	2.70	0.100274177
SNP05_256	**Pv05**	104.97	0.0072	0.9324	2.07	0.150113621
SNP05_260	**Pv05**	110.55	0.2535	0.6146	2.71	0.099783743
SNP05_265	**Pv05**	112.68	0.0000	1.0000	3.34	0.067586621
SNP05_281	**Pv05**	118.26	0.7042	0.4014	1.67	0.196112932
SNP05_283	**Pv05**	118.96	0.3630	0.5469	2.10	0.146940016
SNP05_284	**Pv05**	119.06	0.3630	0.5469	2.10	0.146940016
SNP05_290	**Pv05**	119.81	0.4706	0.4927	1.36	0.243709934
SNP05_297	**Pv05**	125.06	0.0000	1.0000	0.08	0.780845776
SNP05_332	**Pv05**	128.04	0.0282	0.8667	0.82	0.365076608
SNP05_345	**Pv05**	128.72	0.0657	0.7977	0.72	0.397174128
SNP05_358	**Pv05**	133.92	0.3475	0.5555	0.59	0.443951993
SNP05_363	**Pv05**	137.29	0.1880	0.6646	0.27	0.600459286
SNP05_367	**Pv05**	138.007	0.1825	0.6692	0.14	0.705109399
SNP05_380	**Pv05**	138.69	0.1143	0.7353	0.20	0.656425318
SNP05_390	**Pv05**	138.79	0.1143	0.7353	0.20	0.656425318
SNP06_006	**Pv06**	0	0.0308	0.8608	0.15	0.697011747
SNP06_020	**Pv06**	1.21	0.1143	0.7353	0.26	0.610726051
SNP06_026	**Pv06**	2.89	0.3475	0.5555	0.82	0.365436243
SNP06_035	**Pv06**	3.45	0.0677	0.7948	0.54	0.460568276
SNP06_042	**Pv06**	4.01	0.7353	0.3912	0.23	0.633849476
SNP06_071	**Pv06**	4.56	0.4571	0.4990	0.21	0.646508415
SNP06_127	**Pv06**	5.71	0.1143	0.7353	0.27	0.603803006
SNP06_151	**Pv06**	9.2	0.1143	0.7353	0.82	0.365436243
SNP06_160	**Pv06**	10.34	0.1773	0.6737	0.71	0.398480278
SNP06_163	**Pv06**	10.89	0.2571	0.6121	0.62	0.432577945
SNP06_165	**Pv06**	12.52	0.8705	0.3508	0.09	0.762467444
SNP06_169	**Pv06**	14.15	0.7143	0.3980	0.02	0.874631954
SNP06_183	**Pv06**	18.1	1.5957	0.2065	0.16	0.688861297
SNP06_200	**Pv06**	19.21	3.1727	0.0749	0.78	0.377360619
SNP06_247	**Pv06**	22.01	1.4000	0.2367	1.53	0.215941469
SNP06_259	**Pv06**	23.67	1.4000	0.2367	0.63	0.429051983
SNP06_266	**Pv06**	26.55	0.7246	0.3946	0.32	0.568698226
SNP06_285	**Pv06**	27.68	1.4203	0.2334	0.11	0.738520008
SNP06_300	**Pv06**	28.82	1.4000	0.2367	0.53	0.467064581
SNP06_315	**Pv06**	32.77	0.0647	0.7991	1.44	0.230454249
SNP06_333	**Pv06**	34.44	0.3475	0.5555	1.74	0.187534439
SNP06_366	**Pv06**	38.73	0.0286	0.8658	0.94	0.333530002
SNP06_401	**Pv06**	40.53	0.0000	1.0000	0.41	0.519507916
SNP06_430	**Pv06**	47.75	0.1127	0.7371	0.88	0.348727943
SNP06_460	**Pv06**	52.58	1.0909	0.2963	2.49	0.114691630
SNP06_483	**Pv06**	56.83	0.7042	0.4014	2.45	0.117599197
SNP06_509	**Pv06**	63.57	1.0286	0.3105	1.13	0.287993417
SNP06_536	**Pv06**	65.8	0.2687	0.6042	1.05	0.304440867
SNP06_560	**Pv06**	66.47	0.2535	0.6146	0.47	0.494328039
SNP06_562	**Pv06**	67.57	0.5827	0.4452	1.14	0.285034718
SNP06_563	**Pv06**	68.66	0.3577	0.5498	1.32	0.251431067

SNP06_582	**Pv06**	71.44	0.2535	0.6146	0.47	0.494328039
SNP06_612	**Pv06**	76.01	1.0286	0.3105	2.31	0.128714270
SNP06_625	**Pv06**	77.09	1.0286	0.3105	2.31	0.128714270
SNP06_644	**Pv06**	79.27	0.0657	0.7977	2.06	0.151266634
SNP06_660	**Pv06**	81.05	0.3577	0.5498	0.47	0.492209006
SNP06_693	**Pv06**	85.28	0.0070	0.9334	0.59	0.443951993
SNP06_700	**Pv06**	85.81	0.3427	0.5583	0.16	0.688088212
SNP06_702	**Pv06**	86.86	0.4507	0.5020	0.44	0.506287477
SNP06_728	**Pv06**	90.11	0.3525	0.5527	0.27	0.603146648
SNP07_001	**Pv07**	0	2.0496	0.1522	0.20	0.653427233
SNP07_004	**Pv07**	2.96	0.2609	0.6095	0.69	0.406400100
SNP07_010	**Pv07**	14.75	0.1176	0.7316	0.04	0.843299719
SNP07_011	**Pv07**	16.4	0.1852	0.6670	0.86	0.353052541
SNP07_021	**Pv07**	17.43	0.1176	0.7316	0.23	0.631826641
SNP07_026	**Pv07**	20.35	0.4638	0.4959	0.09	0.761892288
SNP07_052	**Pv07**	23.62	0.1159	0.7335	0.20	0.658043527
SNP07_073	**Pv07**	25.08	0.0072	0.9324	0.07	0.798557650
SNP07_092	**Pv07**	28.75	0.0629	0.8019	0.32	0.573617647
SNP07_107	**Pv07**	31.23	0.2609	0.6095	0.31	0.578193997
SNP07_125	**Pv07**	34.53	0.3525	0.5527	0.21	0.648545068
SNP07_146	**Pv07**	36.88	1.1077	0.2926	0.10	0.750769649
SNP07_164	**Pv07**	39.46	0.2609	0.6095	0.00	0.994830237
SNP07_175	**Pv07**	43.03	0.2609	0.6095	0.16	0.687888851
SNP07_188	**Pv07**	45.7	0.0667	0.7963	0.28	0.594325037
SNP07_204	**Pv07**	50.24	1.5957	0.2065	0.00	0.952762474
SNP07_222	**Pv07**	52.5	0.2769	0.5987	0.06	0.804702322
SNP07_233	**Pv07**	54.89	1.4203	0.2334	0.18	0.671233514
SNP07_240	**Pv07**	56.09	1.0141	0.3139	0.27	0.605098776
SNP07_243	**Pv07**	57.14	0.7143	0.3980	0.17	0.681124685
SNP07_247	**Pv07**	63.26	0.1159	0.7335	0.01	0.929146485
SNP07_271	**Pv07**	64.43	0.0000	1.0000	0.16	0.690207584
SNP07_286	**Pv07**	67.58	0.2609	0.6095	0.64	0.423962158
SNP07_296	**Pv07**	72.84	0.2535	0.6146	0.48	0.489257110
SNP07_305	**Pv07**	74.14	0.0638	0.8005	0.43	0.510377274
SNP07_312	**Pv07**	74.77	0.0282	0.8667	0.36	0.548087405
SNP07_313	**Pv07**	75.33	0.0000	1.0000	0.94	0.333500537
SNP07_316	**Pv07**	84.91	0.8582	0.3543	0.05	0.829421776
SNP07_317	**Pv07**	89.72	3.8613	0.0494	0.16	0.688236685
SNP08_001	**Pv08**	0	0.5827	0.4452	5.06	0.024547231
SNP08_010	**Pv08**	0.72	1.1986	0.2736	4.75	0.029273887
SNP08_017	**Pv08**	5.63	1.5734	0.2097	0.45	0.504420721
SNP08_027	**Pv08**	9.36	0.3475	0.5555	0.00	0.977529155
SNP08_035	**Pv08**	11.03	0.2647	0.6069	0.08	0.771380917
SNP08_048	**Pv08**	16.2	0.3525	0.5527	0.21	0.646508415
SNP08_057	**Pv08**	17.04	0.0647	0.7991	0.10	0.752681953
SNP08_061	**Pv08**	20.79	0.0000	1.0000	0.48	0.487611369
SNP08_065	**Pv08**	23.11	0.5827	0.4452	0.34	0.560361223
SNP08_106	**Pv08**	25.66	0.1908	0.6622	0.75	0.385761211
SNP08_154	**Pv08**	32.65	0.0000	1.0000	0.24	0.625408041
SNP08_202	**Pv08**	35.83	0.8582	0.3543	0.21	0.646508415
SNP08_221	**Pv08**	38.91	1.1986	0.2736	1.10	0.293949556

SNP08_237	**Pv08**	46.33	2.0496	0.1522	0.76	0.384838232
SNP08_249	**Pv08**	49.4	3.1727	0.0749	1.23	0.267073258
SNP08_260	**Pv08**	55.46	2.8986	0.0887	0.78	0.376570896
SNP08_267	**Pv08**	60.62	1.8028	0.1794	1.14	0.284677770
SNP08_274	**Pv08**	64.28	0.7042	0.4014	0.03	0.874088217
SNP08_277	**Pv08**	67.61	0.8582	0.3543	0.01	0.926384812
SNP08_280	**Pv08**	70.11	0.8462	0.3576	0.08	0.778834273
SNP08_295	**Pv08**	72.94	1.1986	0.2736	0.04	0.837592410
SNP08_304	**Pv08**	73.66	1.2158	0.2702	0.05	0.826944500
SNP08_328	**Pv08**	76.71	0.9237	0.3365	0.01	0.936135719
SNP08_335	**Pv08**	77.7	2.3824	0.1227	0.08	0.771886553
SNP08_355	**Pv08**	80.88	2.5971	0.1071	0.29	0.588203735
SNP08_395	**Pv08**	84.07	1.0746	0.2999	0.09	0.762060919
SNP08_404	**Pv08**	92.13	0.7463	0.3877	0.03	0.869647871
SNP08_431	**Pv08**	96.64	0.2769	0.5987	0.18	0.667455041
SNP08_440	**Pv08**	98.82	0.3740	0.5408	0.01	0.912458841
SNP08_447	**Pv08**	103.2	0.0657	0.7977	0.86	0.352407403
SNP08_450	**Pv08**	104.6	0.3577	0.5498	1.16	0.280507763
SNP08_457	**Pv08**	110.24	1.6187	0.2033	0.71	0.400878465
SNP08_464	**Pv08**	110.94	0.7246	0.3946	0.81	0.367277762
SNP08_484	**Pv08**	115.04	0.8963	0.3438	0.47	0.493048629
SNP08_490	**Pv08**	115.85	0.8705	0.3508	0.56	0.453222088
SNP08_496	**Pv08**	118.64	3.5588	0.0592	0.22	0.640779298
SNP08_500	**Pv08**	120.91	2.0791	0.1493	0.09	0.768880142
SNP08_504	**Pv08**	124.69	0.2647	0.6069	0.00	0.994867858
SNP08_511	**Pv08**	127.36	0.0286	0.8658	0.06	0.811401823
SNP08_528	**Pv08**	129.86	0.2535	0.6146	0.18	0.673655211
SNP08_547	**Pv08**	135.16	0.4571	0.4990	0.45	0.500578489
SNP08_560	**Pv08**	140.41	0.5827	0.4452	0.45	0.500578489
SNP08_583	**Pv08**	143.78	0.0286	0.8658	1.09	0.296825711
SNP08_596	**Pv08**	146.44	0.4638	0.4959	0.52	0.471727673
SNP08_604	**Pv08**	150.69	0.1159	0.7335	0.55	0.458784101
SNP08_611	**Pv08**	153.23	0.8705	0.3508	0.24	0.621746993
SNP08_622	**Pv08**	157.46	2.3143	0.1282	0.21	0.645186186
SNP08_633	**Pv08**	159.74	1.8551	0.1732	1.08	0.299770395
SNP08_639	**Pv08**	164.87	0.2571	0.6121	0.23	0.630234012
SNP08_643	**Pv08**	166.48	0.0000	1.0000	0.34	0.559852010
SNP08_652	**Pv08**	167.6	0.0657	0.7977	0.36	0.549140133
SNP08_670	**Pv08**	171.89	0.1825	0.6692	0.64	0.423962158
SNP08_685	**Pv08**	173.63	0.1908	0.6622	1.78	0.181733667
SNP08_726	**Pv08**	178.28	0.0294	0.8638	1.24	0.266328299
SNP08_754	**Pv08**	181.13	0.0071	0.9329	0.87	0.352134950
SNP08_785	**Pv08**	184.94	0.0286	0.8658	0.66	0.417058946
SNP09_009	**Pv09**	0	0.7042	0.4014	0.48	0.489677805
SNP09_023	**Pv09**	0.68	1.1818	0.2770	0.76	0.383983530
SNP09_049	**Pv09**	3.39	0.4507	0.5020	0.32	0.570226727
SNP09_076	**Pv09**	4.09	0.5745	0.4485	0.40	0.527112313
SNP09_098	**Pv09**	6.35	0.0629	0.8019	0.41	0.519507916
SNP09_119	**Pv09**	10.08	0.0071	0.9329	0.52	0.469011610
SNP09_129	**Pv09**	11.46	0.0282	0.8667	0.71	0.398480989
SNP09_137	**Pv09**	14.96	0.0070	0.9334	0.21	0.646320615

SNP09_158	**Pv09**	15.71	0.0290	0.8648	0.16	0.692413287
SNP09_178	**Pv09**	18.74	0.1799	0.6715	0.00	0.944172907
SNP09_191	**Pv09**	22.52	0.0071	0.9329	0.07	0.794627227
SNP09_207	**Pv09**	25.95	0.5912	0.4419	0.33	0.564831288
SNP09_221	**Pv09**	29.03	0.4638	0.4959	0.10	0.749199514
SNP09_228	**Pv09**	33.47	1.0435	0.3070	0.10	0.750831884
SNP09_249	**Pv09**	33.57	1.9692	0.1605	0.10	0.755662584
SNP09_255	**Pv09**	35.1	3.1727	0.0749	0.35	0.553082441
SNP09_260	**Pv09**	42.09	3.1727	0.0749	0.16	0.688260587
SNP09_271	**Pv09**	45.57	2.9851	0.0840	0.88	0.347192813
SNP09_275	**Pv09**	46.35	3.5588	0.0592	0.91	0.339668816
SNP09_279	**Pv09**	49.52	3.6119	0.0574	2.00	0.156994666
SNP09_288	**Pv09**	52.72	1.8551	0.1732	1.45	0.228686259
SNP09_294	**Pv09**	59.42	3.5072	0.0611	0.25	0.615405973
SNP09_302	**Pv09**	63.21	3.9185	0.0478	0.20	0.654688356
SNP09_313	**Pv09**	63.31	3.5588	0.0592	0.14	0.710940313
SNP09_317	**Pv09**	69.25	3.8613	0.0494	0.01	0.917525259
SNP09_323	**Pv09**	71.54	3.2667	0.0707	0.06	0.811334372
SNP09_328	**Pv09**	77.69	3.9185	0.0478	0.00	1.000000000
SNP09_329	**Pv09**	91.9	3.8613	0.0494	1.88	0.170706614
SNP09_331	**Pv09**	92.66	3.5588	0.0592	1.60	0.206261887
SNP09_335	**Pv09**	94.19	3.8613	0.0494	1.47	0.225442317
SNP09_339	**Pv09**	96.5	3.5588	0.0592	1.04	0.306950333
SNP09_341	**Pv09**	118.62	3.5072	0.0611	0.77	0.381414330
SNP09_342	**Pv09**	120.59	3.3664	0.0665	0.06	0.814548751
SNP09_345	**Pv09**	130.12	2.9412	0.0863	0.16	0.686167850
SNP09_346	**Pv09**	130.22	2.9851	0.0840	0.76	0.382153442
SNP09_348	**Pv09**	141.18	1.5957	0.2065	0.20	0.653427233
SNP09_352	**Pv09**	145.26	2.8986	0.0887	0.09	0.758946456
SNP09_355	**Pv09**	146.81	1.2519	0.2632	0.01	0.911759263
SNP09_357	**Pv09**	151.26	1.8286	0.1763	0.72	0.397568197
SNP09_362	**Pv09**	152	1.6917	0.1934	0.28	0.598739291
SNP09_365	**Pv09**	154.08	1.0588	0.3035	0.52	0.472303514
SNP09_373	**Pv09**	156.86	2.3143	0.1282	1.25	0.262989438
SNP09_378	**Pv09**	158.94	1.1986	0.2736	2.01	0.156732756
SNP09_393	**Pv09**	161.24	0.4571	0.4990	2.18	0.140216762
SNP09_410	**Pv09**	161.99	0.3525	0.5527	2.93	0.086831525
SNP09_416	**Pv09**	164.29	0.8705	0.3508	0.93	0.335133557
SNP09_423	**Pv09**	166.58	0.2571	0.6121	0.87	0.352134950
SNP09_453	**Pv09**	171.88	0.3427	0.5583	1.90	0.168194426
SNP09_460	**Pv09**	173.25	0.3427	0.5583	0.98	0.321109402
SNP09_464	**Pv09**	176.72	0.4507	0.5020	0.13	0.715425805
SNP10_015	**Pv10**	0	1.2158	0.2702	0.17	0.677232937
SNP10_016	**Pv10**	1.11	0.8705	0.3508	0.22	0.637405020
SNP10_017	**Pv10**	1.11	0.8705	0.3508	0.22	0.637405020
SNP11_001	**Pv11**	0	0.0071	0.9329	0.09	0.769841847
SNP11_005	**Pv11**	3.99	0.0629	0.8019	0.09	0.763356956
SNP11_007	**Pv11**	5.3	0.0282	0.8667	0.56	0.454364610
SNP11_011	**Pv11**	7.4	0.1127	0.7371	0.39	0.531241814
SNP11_014	**Pv11**	18.86	0.5912	0.4419	2.57	0.109049430
SNP11_030	**Pv11**	21.69	0.1212	0.7277	4.09	0.043258675

SNP11_053	**Pv11**	23.81	0.4638	0.4959	1.83	0.175787653
SNP11_065	**Pv11**	27.32	0.6000	0.4386	0.92	0.338711356
SNP11_078	**Pv11**	30.12	0.1773	0.6737	1.82	0.176936668
SNP11_085	**Pv11**	30.84	0.0638	0.8005	2.34	0.125751230
SNP11_092	**Pv11**	32.28	0.1799	0.6715	2.00	0.157628277
SNP11_095	**Pv11**	38.06	0.0000	1.0000	0.89	0.344706027
SNP11_097	**Pv11**	38.16	0.0000	1.0000	0.89	0.344706027
SNP11_108	**Pv11**	38.88	0.0282	0.8667	0.68	0.409750531
SNP11_119	**Pv11**	43.92	0.1773	0.6737	1.12	0.290318270
SNP11_146	**Pv11**	47.52	0.3684	0.5439	4.65	0.031049240
SNP11_156	**Pv11**	49.87	0.4571	0.4990	2.07	0.150113621
SNP11_164	**Pv11**	52.12	0.0657	0.7977	2.01	0.156383146
SNP11_172	**Pv11**	54.42	0.1176	0.7316	3.31	0.068832125
SNP11_178	**Pv11**	58.25	1.6187	0.2033	5.62	0.017706794
SNP11_186	**Pv11**	63.31	2.3824	0.1227	5.64	0.017529537
SNP11_202	**Pv11**	67.43	0.5912	0.4419	5.82	0.015865217
SNP11_219	**Pv11**	72.04	0.1799	0.6715	5.08	0.024150573
SNP11_226	**Pv11**	72.58	0.1852	0.6670	3.36	0.066698112
SNP11_230	**Pv11**	86.17	1.3803	0.2401	0.57	0.450797483
SNP11_232	**Pv11**	86.9	1.0141	0.3139	0.76	0.383303864
SNP11_234	**Pv11**	88.38	0.4507	0.5020	0.76	0.383303864
SNP11_240	**Pv11**	89.86	0.1127	0.7371	1.23	0.268029630
SNP11_285	**Pv11**	91.66	0.3525	0.5527	1.17	0.279500610
SNP11_299	**Pv11**	95.16	0.7246	0.3946	0.44	0.507659186
SNP11_315	**Pv11**	96.72	0.2687	0.6042	0.21	0.643902110
SNP11_336	**Pv11**	97.5	0.6000	0.4386	0.99	0.320677013
SNP11_373	**Pv11**	99.49	0.8705	0.3508	0.11	0.738520008
SNP11_384	**Pv11**	100.48	0.0294	0.8638	0.05	0.827366081
SNP11_391	**Pv11**	103.34	0.9237	0.3365	0.01	0.905385637
SNP11_398	**Pv11**	105.46	1.0588	0.3035	0.10	0.749394185
SNP11_406	**Pv11**	106.93	1.2336	0.2667	0.14	0.703797986
SNP11_410	**Pv11**	107.91	1.0435	0.3070	0.15	0.697451520
SNP11_413	**Pv11**	108.72	1.6423	0.2000	0.36	0.548305724
SNP11_419	**Pv11**	109.53	1.2336	0.2667	0.52	0.471229899
SNP11_428	**Pv11**	111.15	1.2901	0.2560	0.05	0.826759226
SNP11_439	**Pv11**	112.78	1.6187	0.2033	0.15	0.697060256
SNP11_454	**Pv11**	115.82	0.7463	0.3877	0.00	0.950316013
SNP11_465	**Pv11**	116.71	0.4776	0.4895	0.35	0.556671875
SNP11_469	**Pv11**	117.65	0.8963	0.3438	0.70	0.403990598
SNP11_471	**Pv11**	123.05	0.2687	0.6042	0.51	0.475682209
SNP11_475	**Pv11**	124.08	0.2647	0.6069	0.70	0.403990598
SNP11_493	**Pv11**	125.01	0.0657	0.7977	0.70	0.402194822
SNP11_518	**Pv11**	128.09	1.5077	0.2195	0.06	0.809424671
SNP11_539	**Pv11**	132.69	0.0072	0.9324	0.72	0.397174128
SNP11_553	**Pv11**	134.86	0.0647	0.7991	0.04	0.846068523
SNP11_567	**Pv11**	137.75	1.4000	0.2367	0.03	0.858036071
SNP11_587	**Pv11**	138.7	0.8582	0.3543	0.03	0.863617802
SNP11_590	**Pv11**	143.26	1.3803	0.2401	0.00	0.970355548
SNP11_608	**Pv11**	146.09	1.4412	0.2299	0.52	0.472303514
SNP11_615	**Pv11**	146.19	1.5957	0.2065	0.33	0.566187344
SNP11_628	**Pv11**	147.27	1.6187	0.2033	0.37	0.543630385
