# Supplementary material for: Integrating genetic and physical positions of the anthracnose resistance genes described in bean chromosomes Pv01 and Pv04
Source: PLoS One. 2019 Feb 14;14(2):e0212298. doi: 10.1371/journal.pone.0212298 (PMC6375601; doi:10.1371/journal.pone.0212298)
Supplement: S4 Table — A) Tags sequences including the SNPs used in this work. B) Primer sequences of SSR develop for this work (PDF) [file pone.0212298.s005.pdf]

**Table S4**. **Sequences** A) Tags sequences including the SNPs used in this work. B) Primer sequences of SSR develop for this work.

| Marker name | Chromosome | TAG sequences |
|---|---|---|
| **SNP01_351** | Pv01 | CAGCACCACCACTGGATTGGTGGATTGCTCCTCTACCCGAGATGCTGTCACCGCCACAGAATCG |
| **SNP01_353** | Pv01 | CAGCACACGGTTCTGCAAATTTGGTGGGAATTACGTTGAAGGCTAATCATATTGCGGAAGCTGA |
| **SNP01_372** | Pv01 | CAGCGTAAAATCCAATGTCGGCAATTTTGAAGAAAGAAGAACAAGTTTTGTTGTAATCTGGATG |
| **SNP01_390** | Pv01 | CTGCACAGACAGCGACACCTTTTTCATCAACCGGTTCCGTGCTTGGATCAGAGCCTCCTTCTTT |
| **SNP01_404** | Pv01 | CTGCTCGTGTTGAAGGAGCAAGGCAAGAAGCAATGCAATCTGCCCTTGTTGATGTTGTGAAGCA |
| **SNP01_418** | Pv01 | CTGCATGTCTTTACCTCTAAGTCTCAATTATAATCCTTATTTCCCTAATGGACTACCCACATGC |
| **SNP01_435** | Pv01 | CAGCGGAAGAAAACAGCACAATGATTCTATTTCGTCAACATACTTCAAAATAGTTTATAATCTG |
| **SNP01_462** | Pv01 | CAGCCCAGCCACGCACCAGTACGCACCATCACTCGTGCTTCTCCATGCACCGCACACCAGCGCC |
| **SNP01_474** | Pv01 | CTGCAATGTCCAGGATCGTGATCAAAACTGTGTCTGTGACCAAAACTTGAAACCTGGATGGTTA |
| **SNP01_475** | Pv01 | CTGCAGGTTCTCAAGGAGCAACCGTATGTCCCTCCAGGCTCCCGGATTTTCGTCGCGATTCCAT |
| **SNP01_476** | Pv01 | CTGCCACCGCCACTACTCCCGGAAAACGCCGACAACTTACCGTCAAACTTGTTTGCTCCGCCGC |
| **SNP01_477** | Pv01 | CTGCAATTGTTGGGTAGGAGATAGCCAACGACAGTGAAGAAACCATGGAAACACCATTAGCAAA |
| **SNP01_478** | Pv01 | CTGCACTGCTTTCGAAGCTTGCGATAAGGCCCTTCTCTTATGGCCTTGTTGTGGTTTCTGTCTA |
| **SNP01_479** | Pv01 | CAGCCGTCTCTCCCCGGCTACACTCACCGCCGCTTCACTCTCATGCGCTCCTCTCACGTTGAGG |
| **SNP01_480** | Pv01 | CAGCACAAAATACCCAGTCAGACATAGTAGAATGCATTCAAACATGGAACATTATTGAATCTTT |
| **SNP01_481** | Pv01 | CTGCGGTCAATGATGACAAGGATGAAGTTGAAGAATCTCTGCACTATTTATCAAACAAAATTGA |
| **SNP01_482** | Pv01 | CTGCTCAGGACCAAGGGCAATCCGCAGATCCTTCAGAGATGAAAACGCCAGTATATGCACAGAG |
| **SNP01_483** | Pv01 | CAGCGATGGCTGATAAAGGAACGGAAGATCAATACTTGAAACGACGCGTAACTACAGGATGTTA |
| **SNP01_484** | Pv01 | CTGCTGTGATGGGGATTGCCCTCCCTGCTCAGAGGTTCGTGCCTGTTTTTGCAGAAAAAAAAAA |
| **SNP01_485** | Pv01 | CTGCCATACAATAACCCCGCTAGAGAAACGGCAATTTTCTTTTTACCAAGTCTATCAGGTCCAA |
| **SNP01_486** | Pv01 | CTGCTCTTCTCTGTGGATCAACAGCGAAGCCAGGCTCTTGAAGCGCATGCTG |
| **SNP01_487** | Pv01 | CTGCTCTTCTCTGTGGATCAACAGCGAAGCCAGGCTCTTGAAGCGCATGCTG |
| **SNP01_488** | Pv01 | CAGCGACTGACAGGAAAACGACCATGAACTCAAGTGTATACAATGAACTCTAGTGTGTATACAG |
| **SNP01_489** | Pv01 | CTGCTACTTTGCGCTCTGTATACACACTAGAGTTCATTGTATACACTTGAGTTCATGGTCGTTT |
| **SNP01_490** | Pv01 | CAGCTTCCATCACAAGGAAAAGTGGAGTAAATCCATGAAAAACATGAGCCAGGATTCAAAGTCG |
| **SNP01_491** | Pv01 | CAGCGAAAAAATAAGAGAGCCTAGGTAGTGACACGCTTTTATCTTCATGTACTCATATCTGGGT |
| **SNP01_492** | Pv01 | CTGCAGGAGATCCTTCAAAATTAGCTAACTGAATGCCGCTAACAACAAGAACAAAATGCCTAAA |
| **SNP01_493** | Pv01 | CAGCATAAATGACAGCCTCGTACTTGCTTCCATGCAACCTCTTGTTCTCATAACGAAGTGGTGC |
| **SNP01_494** | Pv01 | CTGCAAAGCAGAGGAATGGTGGCGGCTTGGACCGGCGTGCAGAAGTCGAAGCCAGATTGTGATA |
| **SNP01_502** | Pv01 | CTGCTGAGGAGGCTCTGAGGGGATGGATTCCAGAGGATGACCTCAGAAGGTATAACACTATCAA |
| **SNP01_502** | Pv01 | CTGCTGAGGAGGCTCTGAGGGGATGGATTCCAGAGGATGACCTCAGAAGGTATAACACTATCAA |
| **SNP01_543** | Pv01 | CAGCTTATAAAACAAGGAAAGGGATTTGATGTGCCAGGAGGAGACAAAGGCAATGATGAAGAAA |
| **SNP01_562** | Pv01 | CAGCTTGTGGGAGAAGGTTGTGGCGTTTTGGGGTTTAGAGATGCTCAAGGGTGCACTGTTTTGC |
| **SNP01_572** | Pv01 | CTGCAATCTTCACAAATTCCTCCAAAGTAACTCCTGCAACATCAAGCACACAAAGTACGACCCC |
| **SNP01_582** | Pv01 | CTGCGGCATGTTCTATTTAATTGGTTAAGTTTTGGTTAATGGTAATTTTTGTATGTTGCGTTCT |
| **SNP01_606** | Pv01 | CAGCTAAATTCCCCTGAGTAAAAGACAGCAATTCTTATAAAGCACAATCAAAATTACAGAAGGA |
| **SNP04_020** | Pv04 | CAGCACCTTTGATGAACTTGGACAGGGTCTTTATTTGGATGGAAAGTTAAATGGTGATTTTAGA |
| **SNP04_021** | Pv04 | CAGCACAAGGGAGAGTAGACTGGAACATATCCCAATCTGGCTGGATGAAGTTGCCCATCCATAA |
| **SNP04_022** | Pv04 | CAGCCTCAAGAGCCATATCTGGTGGCCCAATTTACATCAGTGACACAGTTGGGAACCACAACTT |
| **SNP04_023** | Pv04 | CTGCAGTTGTCCTCAGAAAACATCAACAACACCAAACAATGTCGAGCAAAGACTCAAAGGTGAA |
| **SNP04_024** | Pv04 | CTGCAGTTGTCTTCAGAAAACATCAACAACACCAAACAATGTCGAGCAAAGACTCAAAGGCGAA |
| **SNP04_025** | Pv04 | CTGCAGTTGTCCTCAGAAAACATCAACAACACCAAACAATGTCGAGCAAAGACTCAAAGGTGAA |
| **SNP04_026** | Pv04 | CTGCAAGTTGCAAAGCTACAAATGTCCAATACGAATCAGAAACCAGAAGCTCAATCTAGCACCA |
| **SNP04_027** | Pv04 | CTGCTTGCCCACGAGGAGTTACAATCACGCCATCCCCAGTGTTGGTACTATCACTGCGAATGCT |

| | | |
|---|---|---|
| **SNP04_028** | Pv04 | CAGCGGAGCTCGATGGCCTCTCATACTGGGTGACATTGGTATCGGGATTCCAATAGTAAAGATA |
| **SNP04_029** | Pv04 | CAGCGGAGGTCGATGGCCTCTCATACTGGGTGACATTGGTCTCGGGATTCCAATAGTAAAGATA |
| **SNP04_030** | Pv04 | CAGCAGAACTTGTAGGTGGTACTCTTCTTTCGGCCTTTCTTCAGGTTGCATTCGACAGGCTCGC |
| **SNP04_031** | Pv04 | CAGCAGAACTTGTAGGTGGTACTCTTCTTTCGGCCTTTCTTCAGGTTGCATTCGACAGGCTCGC |
| **SNP04_032** | Pv04 | CAGCATATTCCAACATATGAACGGGGATGCCGAGGATAACATGCCACCTAAGCGACAACATATG |
| **SNP04_034** | Pv04 | CAGCAACTTTTGTTTGCAAAAGTGGCAATTAAATTATCTGTAATTTTCACCTCTGAGCAGGGGC |
| **SNP04_042** | Pv04 | CAGCGGAAGGGACCAGACTTATTTGATAAAGGTGGTGCAGATGTAAAAAGCGCTGGAAGCGGGA |
| **SNP04_047** | Pv04 | CAGCCTGTAATTTAAAAATGACATGCAAGAAAACGGCTATTATCAGACATATATGTATGGTCCA |
| **SNP04_054** | Pv04 | CAGCGGCGACTTCATGAACCACATTCCCAGGTCCACCATGGGCCCCACCGCCGTCGCTTGCAGA |
| **SNP04_766059** | Pv04 | CTGCGGATAACTTGTTGAATTGTTTTTATGCAGATATGCTATTTTTTCTATAAAAACATTGCGT |
| **SNP04_766107** | Pv04 | CTGCGGATAACTTGTTGAATTGTTTTTATGCAGATATGCTATTTTTTCTATAAAAACATTGCGT |
| **SNP04_1023546** | Pv04 | CTGCATTCGTCCGCAGTTTGTTAGGACTAACTGCTGGGTTTGGGTTTTGGAGTCATTTCAACCT |
| **SNP04_1058721** | Pv04 | CAGCAAACAAAAATAATCTTGTTAATGAAAGGGAAAAACAGCAGTGAGGACAGTTTCTAAGAAA |
| **SNP04_1058766** | Pv04 | CAGCTAGCTGGGGATGCATTGGTGGATGATGATGAATTACAGGACCTTTCAGAGAAGGAAGAAA |
| **SNP04_1058798** | Pv04 | CAGCTAGCTGGGGATGCATTGGTGGATGATGATGAATTACAGGACCTTTCAGAGAAGGAAGAAA |
| **SNP04_1104475** | Pv04 | CTGCCCTTAGCATCCGGTTCTGAAGTTCTGACTCACTCAGAGCTTTTACGGTGATTACCGTTCG |
| **SNP04_1104710** | Pv04 | CTGCTTAGTGGGAGTGAGAAGAAGTTACCGTCCATTTCGACGATGGGATTGAGAACGGGAAGTC |
| **SNP04_1154798** | Pv04 | CAGCACTGCTAACTGACCAAGTCTGACAGACAGGTGTAAATAACAAACTAACAGAATACAATTG |
| **SNP04_1214395** | Pv04 | CTGCGCCTGTCTCCATCACTTCCATGTTCTCCACCATCCTCTACCGATCCATCCAAACTAAAAA |
| **SNP04_1214415** | Pv04 | CTGCGCCTGTCTCCATCACTTCCATGTTCTCCACCATCCTCTACCGATCCATCCAAACTAAAAA |
| **SNP04_1216747** | Pv04 | CTGCGGTTCTGCAATATCCTCCAGTTTATATGGGTAGGCTTTTTGACAGCTCAAATTACCAAAC |
| **SNP04_1216789** | Pv04 | CTGCGGTTCTGCAATATCCTCCAGTTTATATGGGTAGGCTTTTTGACAGCTCAAATTACCAAAC |
| **SNP04_1216801** | Pv04 | CAGCTCAAATTACCAAACCGAGGAAGATGATGGGAAAAAGGTTATTGATCCAAAGAAAATCCAG |
| **SNP04_1231633** | Pv04 | CAGCAGTGAAGAACAGAGAAATTGAACTAAAAAAACACTGATATGATTAACAGAAGGAAGAGGT |
| **SNP04_1308175** | Pv04 | CAGCATCCATTGAGAGGAAGGAATTCCAAAGCTTTACCTTAAGGGGGTTTCAAATTTCAAAAAG |
| **SNP04_1404668** | Pv04 | CAGCCAGGATTCATGTTAAAGGTAAGAAAAGCATTATGGTGGCCAATGTAAATAATCATTTTAT |
| **SNP04_1404684** | Pv04 | CAGCCAGGATTCATGTTAAAGGTAAGAAAAGCATTATGGTGGCCAATGTAAATAATCATTTTAT |
| **SNP04_1481068** | Pv04 | CAGCTAGCATTCCTTGGCACTTGAGACTGCCTTTGAAGGTTTTGAGCATGCTCAGTTTTCTGCT |
| **SNP04_1485253** | Pv04 | CAGCTTCAACAAAGCTCAAATTGGAAGGTTTGTGAGCCAATAACTTTTCTTCAACAGCAGTATA |
| **SNP04_1485455** | Pv04 | CTGCGGGGAACTAAGACTGTTCCGGGGTATGATGTTGCTGGTGTGGTGGTGAGTGTGGGAAGTC |
| **SNP04_1521374** | Pv04 | CAGCATAAATAATTAAACATTCACTAACACACATTTTTTGAGGATGTGTAGTCGAGTGGGGTAA |
| **SNP04_1539131** | Pv04 | CAGCAGTAGCATTGAAATCCTTGACTTCCAAGTCCTCAAGATTAGGACTCCCAGAAAGAAACTG |
| **SNP04_1539146** | Pv04 | CAGCAGTAGCATTGAAATCCTTGACTTCCAAGTCCTCAAGATTAGGACTCCCAGAAAGAAACTG |
| **SNP04_1854518** | Pv04 | CAGCAAAGATGTCATAGCAGTTGATCCATACTGTTCCAACTCGCAATGCCCGCATCAAGGTATT |

**Table S4**. B) Primer sequences of SSR develop for this work.

| Marker name | Chromosome | Prmer Forward seq | Primer Revers seq |
|---|---|---|---|
| **SSR4_1.647.8** | Pv04 | CCTGTTTTGTGTTGTTTTAGG | ATGAAAAGTACAGGCACCATA |
| **SSR4_1.743.4** | Pv04 | CCCCATAGGTTTAAGGTAAAG | TTACAGAAAAAGGCATAGACG |
| **SSR4_1.669.3** | Pv04 | AACAAACGAAAATGGAACTT | AATTGATGTGCATACCATGAGA |
| **SSR4_1.612.8** | Pv04 | GAAAGGATGCGAATTATTACA | TGTTGACTGTTTCTGATACGA |
