# Supplementary material for: Integrating genetic and physical positions of the anthracnose resistance genes described in bean chromosomes Pv01 and Pv04
Source: PLoS One. 2019 Feb 14;14(2):e0212298. doi: 10.1371/journal.pone.0212298 (PMC6375601; doi:10.1371/journal.pone.0212298)
Supplement: S5 Table — A/ List of annotated genes found for the bounded region in the chromosome Pv01 from the G19833 genome. Red markers did not show recombination with the resistance locus to race 65 (see Fig 1) b/ List of annotated genes found for the bounded region in the chromosome Pv01 from the BAT93 genome. Red markers did not show recombination with the resistance locus to race 65. c/ List of annotated genes found for the bounded region in the chromosome Pv04 from the G19833 genome. Red markers did not show recombination with the resistance locus to race 38. * Functional annotation related to pathogen resistance. d/ List of annotated genes found for the bounded region in the chromosome Pv04 from the BAT93 genome. Red markers did not show recombination with the resistance locus to race 38. (PDF) [file pone.0212298.s006.pdf]

S5 Table List of annotated genes in the bounded regions obtained from the G19833 genome [46] https://phytozome.jgi.doe.gov/pz/portal.html) and BAT93 genome (47; https://genomevolution.org/coge/). * Functional annotation related to pathogen resistance

A/ List of annotated genes found for the bounded region in the chromosome Pv01 from the G19833 genome. Red markers did not show recombination with the resistance locus to race 65 (see Figure 1) * Functional annotation related to pathogen resistance

Marker loci	Chromosome	Start pos.	End Pos	Annotated genes	
IND01_502219/ SNP01_483	Chr01	49508873	49512603	Phvul.001G242600	
	Chr01	49523850	49527672	Phvul.001G242700	
	Chr01	49529546	49533284	Phvul.001G242800	
	Chr01	49536312	49537058	Phvul.001G242900	
	Chr01	49537487	49540675	Phvul.001G243000	
	Chr01	49543248	49549140	Phvul.001G243100	
	Chr01	49547904	49557096	Phvul.001G243200	
	Chr01	49556457	49563883	Phvul.001G243300	
	Chr01	49560638	49573698	Phvul.001G243400	
	Chr01	49571399	49573717	Phvul.001G243500	*
	Chr01	49575989	49578453	Phvul.001G243600	*
	Chr01	49578205	49579934	Phvul.001G243700	*
	Chr01	49582230	49586451	Phvul.001G243800	*
	Chr01	49613454	49618830	Phvul.001G243900	
	Chr01	49628276	49631721	Phvul.001G244000	
	Chr01	49637056	49639871	Phvul.001G244100	
	Chr01	49642087	49644707	Phvul.001G244200	
SNP01_490	Chr01	49646711	49658925	Phvul.001G244300	
	Chr01	49660144	49662487	Phvul.001G244400	
	Chr01	49667176	49669090	Phvul.001G244500	
	Chr01	49678315	49679803	Phvul.001G244600	
	Chr01	49688511	49689918	Phvul.001G244700	
	Chr01	49698853	49700855	Phvul.001G244800	
	Chr01	49707470	49715399	Phvul.001G244900	
	Chr01	49716447	49721102	Phvul.001G245000	
SNP01_491	Chr01	49722532	49725081	Phvul.001G245100	

b/ List of annotated genes found for the bounded region in the chromosome Pv01 from the BAT93 genome. Red markers did not show recombination with the resistance locus to race 65.

Marker loci	Chromosome		Start pos.	End Pos	Annotated genes
SNP01_491	Pv01	scaffold00007	1719097	1722515	PHASIBEAM10F002189
	Pv01	scaffold00007	1723549	1728189	PHASIBEAM10F002190
	Pv01	scaffold00007	1729227	1733911	PHASIBEAM10F002191
	Pv01	scaffold00007	1733935	1736610	PHASIBEAM10F002192
	Pv01	scaffold00007	1743894	1746162	PHASIBEAM10F002193
	Pv01	scaffold00007	1754379	1757121	PHASIBEAM10F002194
	Pv01	scaffold00007	1767209	1767758	PHASIBEAM10F002195
	Pv01	scaffold00007	1780024	1782861	PHASIBEAM10F002196
	Pv01	scaffold00007	1786237	1788546	PHASIBEAM10F002197
SNP01_490	Pv01	scaffold00007	1789721	1801988	PHASIBEAM10F002198
	Pv01	scaffold00007	1803954	1806605	PHASIBEAM10F002199
	Pv01	scaffold00007	1808622	1811705	PHASIBEAM10F002200
	Pv01	scaffold00007	1816545	1820262	PHASIBEAM10F002201
	Pv01	scaffold00007	1829158	1834768	PHASIBEAM10F002202
	Pv01	scaffold00007	1862445	1865632	PHASIBEAM10F002203
	Pv01	scaffold00007	1868807	1870036	PHASIBEAM10F002204
	Pv01	scaffold00007	1870389	1871495	PHASIBEAM10F002205
	Pv01	scaffold00007	1874846	1876869	PHASIBEAM10F002206
	Pv01	scaffold00007	1877556	1884871	PHASIBEAM10F002207
	Pv01	scaffold00007	1885813	1890815	PHASIBEAM10F002208
	Pv01	scaffold00007	1892793	1899046	PHASIBEAM10F002209
	Pv01	scaffold00007	1899916	1904290	PHASIBEAM10F002210
	Pv01	scaffold00007	1907264	1910443	PHASIBEAM10F002211
	Pv01	scaffold00007	1911140	1911532	PHASIBEAM10F002212
	Pv01	scaffold00007	1914749	1918541	PHASIBEAM10F002213
	Pv01	scaffold00007	1920362	1924818	PHASIBEAM10F002214
SNP01_483	Pv01	scaffold00007	1937154	1937217	-
IND01_502219	Pv01	scaffold00007	1937873	1940926	PHASIBEAM10F002215

c/ List of annotated genes found for the bounded region in the chromosome Pv04 from the G19833 genome. Red markers did not show recombination with the resistance locus to race 38. * Functional annotation related to pathogen resistance

Marker loci	Chromosome	Start pos.	End Pos	Annotated genes	
SNP04_766107	Pv04	1022919	1022975	-	
	Pv04	1042615	1043470	Phvul.004G009000	
	Pv04	1043536	1044075	Phvul.004G009100	*
	Pv04	1047155	1058973	Phvul.004G009118	
	Pv04	1047155	1051761	Phvul.004G009109	
	Pv04	1057389	1058973	Phvul.004G009127	
	Pv04	1063726	1067743	Phvul.004G009136	*
	Pv04	1069859	1071418	Phvul.004G009145	
	Pv04	1103140	1118146	Phvul.004G009154	*
	Pv04	1129267	1132629	Phvul.004G008900	*
	Pv04	1137597	1139195	Phvul.004G008909	*
	Pv04	1146369	1149704	Phvul.004G008918	*
	Pv04	1154354	1155845	Phvul.004G008927	
	Pv04	1156680	1160125	Phvul.004G009300	*
	Pv04	1168639	1169717	Phvul.004G009400	
	Pv04	1182525	1185746	Phvul.004G009509	
	Pv04	1182525	1185746	Phvul.004G009509	
	Pv04	1191387	1192524	Phvul.004G009518	
	Pv04	1203535	1207848	Phvul.004G009527	*
	Pv04	1208910	1217117	Phvul.004G009536	
	Pv04	1228652	1233028	Phvul.004G009800	*
	Pv04	1237533	1241552	Phvul.004G009809	
	Pv04	1242901	1255265	Phvul.004G009818	
	Pv04	1262367	1264201	Phvul.004G010100	
	Pv04	1273274	1273678	Phvul.004G010300	
	Pv04	1278226	1282234	Phvul.004G010400	*
SNP04_1023546	Pv04	1282863	1287403	Phvul.004G010500	
	Pv04	1288407	1292222	Phvul.004G010600	
	Pv04	1298498	1299920	Phvul.004G010700	
	Pv04	1315291	1319926	Phvul.004G010800	
	Pv04	1320790	1324143	Phvul.004G010900	
	Pv04	1359319	1361236	Phvul.004G012400	
	Pv04	1370763	1375537	Phvul.004G012500	
	Pv04	1385799	1398207	Phvul.004G012600	*
SNP04_1308175	Pv04	1418845	1427300	Phvul.004G012700	
	Pv04	1429437	1433859	Phvul.004G012801	*
	Pv04	1434735	1440120	Phvul.004G012900	*
	Pv04	1450903	1456425	Phvul.004G013000	*
	Pv04	1461865	1466462	Phvul.004G013100	*
	Pv04	1482045	1486960	Phvul.004G013200	*
	Pv04	1489645	1493416	Phvul.004G013300	*
	Pv04	1506401	1512428	Phvul.004G013350	*
SNP04_1404668	Pv04	1513674	1513737	-	
	Pv04	1520430	1521605	Phvul.004G013400	
	Pv04	1533264	1538109	Phvul.004G013500	
	Pv04	1541722	1547914	Phvul.004G013600	
	Pv04	1550250	1557248	Phvul.004G013700	
	Pv04	1560239	1565500	Phvul.004G013800	
	Pv04	1568934	1572157	Phvul.004G013900	
	Pv04	1575418	1579229	Phvul.004G014000	
	Pv04	1587289	1590156	Phvul.004G014100	
	Pv04	1591447	1593823	Phvul.004G014200	

	Pv04	1596471	1600377	Phvul.004G014300	
	Pv04	1601551	1604610	Phvul.004G014400	
	Pv04	1609805	1616040	Phvul.004G014500	
	Pv04	1616741	1619302	Phvul.004G014600	
	Pv04	1623023	1623747	Phvul.004G014666	
	Pv04	1624174	1625104	Phvul.004G014732	
	Pv04	1627545	1630407	Phvul.004G014800	
	Pv04	1636927	1637452	Phvul.004G014900	
	Pv04	1642019	1643323	Phvul.004G015000	*
	Pv04	1647932	1649255	Phvul.004G015200	
	Pv04	1667777	1668419	Phvul.004G015300	
	Pv04	1671781	1673099	Phvul.004G015400	
	Pv04	1675251	1676448	Phvul.004G015500	
	Pv04	1678112	1683180	Phvul.004G015600	*
	Pv04	1685085	1687198	Phvul.004G015666	
	Pv04	1687342	1689053	Phvul.004G015732	*
	Pv04	1718700	1734046	Phvul.004G015800	*
	Pv04	1726367	1726954	Phvul.004G015900	*
	Pv04	1735410	1741124	Phvul.004G016000	*
	Pv04	1749778	1755565	Phvul.004G016200	
	Pv04	1759056	1760529	Phvul.004G016300	*
	Pv04	1769087	1773138	Phvul.004G016400	*
	Pv04	1779848	1780799	Phvul.004G016466	
	Pv04	1795047	1799504	Phvul.004G016532	*
	Pv04	1815378	1820596	Phvul.004G016600	*
	Pv04	1834565	1836925	Phvul.004G016700	
	Pv04	1839577	1845525	Phvul.004G016800	
	Pv04	1848712	1853596	Phvul.004G016900	*
SSR4_1.743.4	Pv04	1854914	1859100	Phvul.004G017000	
	Pv04	1861229	1862966	Phvul.004G017100	
	Pv04	1871389	1873147	Phvul.004G017200	
	Pv04	1878844	1880098	Phvul.004G017300	
	Pv04	1899660	1904865	Phvul.004G011000	*
IND04_10936	Pv04	1908660	1918334	Phvul.004G011100	
	Pv04	1919514	1928267	Phvul.004G011200	
	Pv04	1934548	1939203	Phvul.004G011300	
	Pv04	1942183	1944366	Phvul.004G011400	
	Pv04	1950872	1953929	Phvul.004G011500	
	Pv04	1955496	1959929	Phvul.004G011600	
	Pv04	1963311	1973902	Phvul.004G011700	
	Pv04	1982817	1986694	Phvul.004G011800	
	Pv04	1988253	1993237	Phvul.004G011900	
	Pv04	2010123	2013813	Phvul.004G012000	
	Pv04	2029514	2034095	Phvul.004G012100	
	Pv04	2034262	2037019	Phvul.004G012200	*
SNP04_1231633	Pv04	2040864	2048272	Phvul.004G012300	
	Pv04	2071770	2081285	Phvul.004G017400	
	Pv04	2103896	2104261	Phvul.004G017450	
	Pv04	2104262	2107197	Phvul.004G017500	
	Pv04	2107717	2121994	Phvul.004G017600	
	Pv04	2130947	2132008	Phvul.004G017700	
	Pv04	2135290	2137901	Phvul.004G017900	
SNP04_1854518	Pv04	2140269	2145007	Phvul.004G018000	

d/ List of annotated genes found for the bounded region in the chromosome Pv04 from the BAT93 genome. Red markers did not show recombination with the resistance locus to race 38.

Marker loci	Chromosome		Start pos.	End Pos	Annotated genes
	Pv04	scaffold00235	5895	10420	PHASIBEAM10F017024
SNP04_1214395 / SNP04_1214415 / SNP04_1216747 / SNP04_1216789 / SNP04_1216801	Pv04	scaffold00235	28002	35874	PHASIBEAM10F017025
	Pv04	scaffold00235	33109	36031	PHASIBEAM10F017026
	Pv04	scaffold00235	73379	75177	PHASIBEAM10F017027
	Pv04	scaffold00235	82539	87404	PHASIBEAM10F017028
	Pv04	scaffold00235	96419	108875	PHASIBEAM10F017029
SNP04_1308175 / SNP04_766107	Pv04	scaffold00235	129837	200730	PHASIBEAM10F017030
SNP04_1404668	Pv04	scaffold00235	227150	227213	-
	Pv04	scaffold00235	247365	252791	PHASIBEAM10F017032
	Pv04	scaffold00235	256453	262660	PHASIBEAM10F017033
	Pv04	scaffold00235	264876	272067	PHASIBEAM10F017034
	Pv04	scaffold00235	274706	280211	PHASIBEAM10F017035
	Pv04	scaffold00235	283354	286679	PHASIBEAM10F017036
	Pv04	scaffold00235	290491	294361	PHASIBEAM10F017037
	Pv04	scaffold00235	301153	304214	PHASIBEAM10F017038
	Pv04	scaffold00235	305341	307834	PHASIBEAM10F017039
	Pv04	scaffold00235	310568	314950	PHASIBEAM10F017040
	Pv04	scaffold00235	316135	319136	PHASIBEAM10F017041
	Pv04	scaffold00235	323552	330014	PHASIBEAM10F017042
	Pv04	scaffold00235	330609	333078	PHASIBEAM10F017043
	Pv04	scaffold00235	336744	339223	PHASIBEAM10F017044
	Pv04	scaffold00235	339990	344304	PHASIBEAM10F017045
	Pv04	scaffold00235	350952	351477	PHASIBEAM10F017046
	Pv04	scaffold00235	514667	517201	PHASIBEAM10F017053
	Pv04	scaffold00235	529494	533386	PHASIBEAM10F017055
SSR4_1.743.4	Pv04	scaffold00235	535003	539443	PHASIBEAM10F017057
	Pv04	scaffold00795	32944	36481	PHASIBEAM10F025884
	Pv04	scaffold00795	32944	51166	PHASIBEAM10F025885
	Pv04	scaffold00795	58818	59279	PHASIBEAM10F025886
	Pv04	scaffold00795	60010	62129	PHASIBEAM10F025887
SNP04_1854518	Pv04	scaffold00795	60583	70283	PHASIBEAM10F025888
	Pv04	scaffold00795	67815	72119	PHASIBEAM10F025889
	Pv04	scaffold00795	86902	93781	PHASIBEAM10F025890
	Pv04	scaffold00795	95948	100792	PHASIBEAM10F025891
	Pv04	scaffold00795	130883	134568	PHASIBEAM10F025894
	Pv04	scaffold00795	139739	143305	PHASIBEAM10F025895
	Pv04	scaffold00719	922	6019	PHASIBEAM10F025438
	Pv04	scaffold00719	7403	11438	PHASIBEAM10F025439
	Pv04	scaffold00719	19882	20301	PHASIBEAM10F025441
	Pv04	scaffold00719	31128	36800	PHASIBEAM10F025442
	Pv04	scaffold00719	37889	41747	PHASIBEAM10F025443

	Pv04	scaffold00719	46398	48607	PHASIBEAM10F025444
	Pv04	scaffold00719	50891	55781	PHASIBEAM10F025445
SNP04_1104475	Pv04	scaffold00719	62654	71367	PHASIBEAM10F025446
IND04_10936	Pv04	scaffold00719	72181	81861	PHASIBEAM10F025447
	Pv04	scaffold00719	85507	91081	PHASIBEAM10F025448
SNP04_1058766 / SNP04_1058798	Pv04	scaffold00719	118120	121454	PHASIBEAM10F025449
IND04_10570	Pv04	scaffold00719	122092	122137	-
